# Bithiophene Imide-Based Self-Assembled Monolayers (SAMs) on NiOx for High-Performance Tin Perovskite Solar Cells Fabricated Using a Two-Step Approach

**DOI:** 10.1021/acsami.4c15688

**Published:** 2024-12-27

**Authors:** Arulmozhi Velusamy, Chun-Hsiao Kuan, Tsung-Chun Lin, Yun-Sheng Shih, Cheng-Liang Liu, De-You Zeng, Yu-Gi Li, Yu-Hao Wang, Xianyuan Jiang, Ming-Chou Chen, Eric Wei-Guang Diau

**Affiliations:** † Department of Chemistry and Research Center of New Generation Light Driven Photovoltaic Modules, National Central University, Taoyuan 32001, Taiwan; ‡ Department of Applied Chemistry and Institute of Molecular Science, National Yang Ming Chiao Tung University, 1001 Ta-Hseuh Road, Hsinchu 300093, Taiwan; § Department of Materials Science and Engineering, National Taiwan University, Taipei 10617, Taiwan; ∥ School of Physical Science and Technology, ShanghaiTech University, Shanghai 201210, China; ⊥ Center for Emergent Functional Matter Science, National Yang Ming Chiao Tung University, 1001 Ta-Hseuh Road, Hsinchu 300093, Taiwan

**Keywords:** bithiophene imide, NiOx, self-assembled monolayer, tin perovskite solar cells, anchoring groups

## Abstract

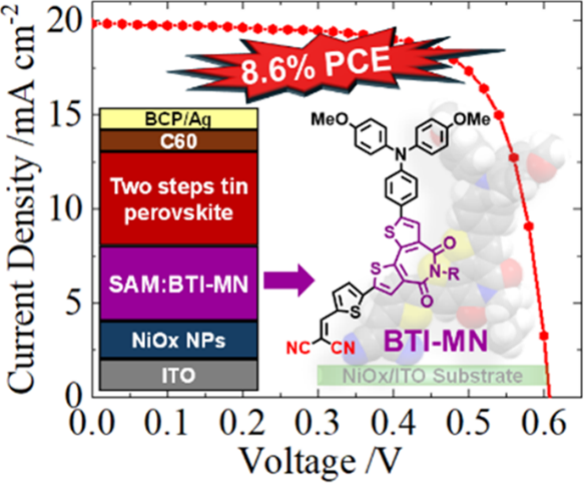

Three new bithiophene imide (BTI)-based organic small molecules, **BTI-MN-b4** (**1**), **BTI-MN-b8** (**2**), and **BTI-MN-b16** (**3**), with varied alkyl side chains, were developed and employed as self-assembled monolayers (SAMs) applied to NiOx films in tin perovskite solar cells (TPSCs). The NiOx layer has the effect of modifying the hydrophilicity and the surface roughness of ITO for SAM to uniformly deposit on it. The side chains of the SAM molecules play a vital role in the formation of a high-quality perovskite layer in TPSCs. The single crystal structure of **BTI-MN-b8** (**2**) was successfully obtained, indicating that a uniform SAM can be formed on the NiOx/ITO substrate with an appropriate size of the alkyl side chain. By combining **BTI-MN-b8** (**2**) with NiOx, a maximum PCE of 8.6% was achieved. The TPSC devices utilizing the NiOx/**BTI-MN-b8** configuration demonstrated outstanding long-term stability, retaining ∼80% of their initial efficiency after 3600 h. Comprehensive characterizations, including thermal, optical, electrochemical, and morphological analyses, alongside photovoltaic evaluation, were carried out thoroughly. This study presents a pioneering strategy for improving TPSC performance, highlighting the efficacy of combining organic SAMs with NiOx as the HTM and offering a promising pathway for future advances in TPSC technology using a two-step fabrication approach.

## Introduction

Over the past few years, there has been widespread agreement that the use of green energy is critical to long-term growth. After decades of research, photovoltaic (PV) technology has achieved significant advances. So far, dye-sensitized solar cells (DSSCs),^1^ organic photovoltaics (OPVs),^2^ and perovskite solar cells (PSCs) have been the primary focus of next-generation solar cell research.^3−6^ PSCs have made remarkable progress by improving their PCE from 3.8%^7^ in 2009 to 26.7%^8^ to date. This rapid progress is mostly owing to perovskite materials’ excellent photoelectric properties, which include a long carrier diffusion length,^9^ a broad light absorption spectrum,^10,11^ minimal exciton binding energy,^12^ high charge transport capacity, and reduced defect density.^13^ Planar PSCs are popular due to their easy preparation and impressive PSC performance.^14−20^ Especially, the inverted p–i–n structure offers several commercialization benefits such as greater stability, fewer hysteresis, and low-temperature processing.^21^ In PSCs, hole transport materials (HTMs) play a crucial role in the disassociation of electron–hole pairs by effectively transporting holes while blocking electrons.^22,23^ Commonly used HTMs in inverted planar PSCs include NiOx,^24−26^ poly[bis(4-phenyl)(2,4,6-trimethylphenyl)amine] (PTAA), and poly(3,4-ethylene dioxythiophene) (PEDOT).^27,28^ PEDOT and NiOx have recently been replaced in PSCs by organic small molecules that self-assemble into a monolayer (SAM) on the ITO surface.^29−32^ Utilizing SAM molecules for PSC offers several advantages, including simple preparation, tunable energy levels, reduced recombination loss, and easy processing.^33−37^ Consequently, there has been significant attention focused on tailoring ITO surface states with suitable SAMs.

**Figure 1:**
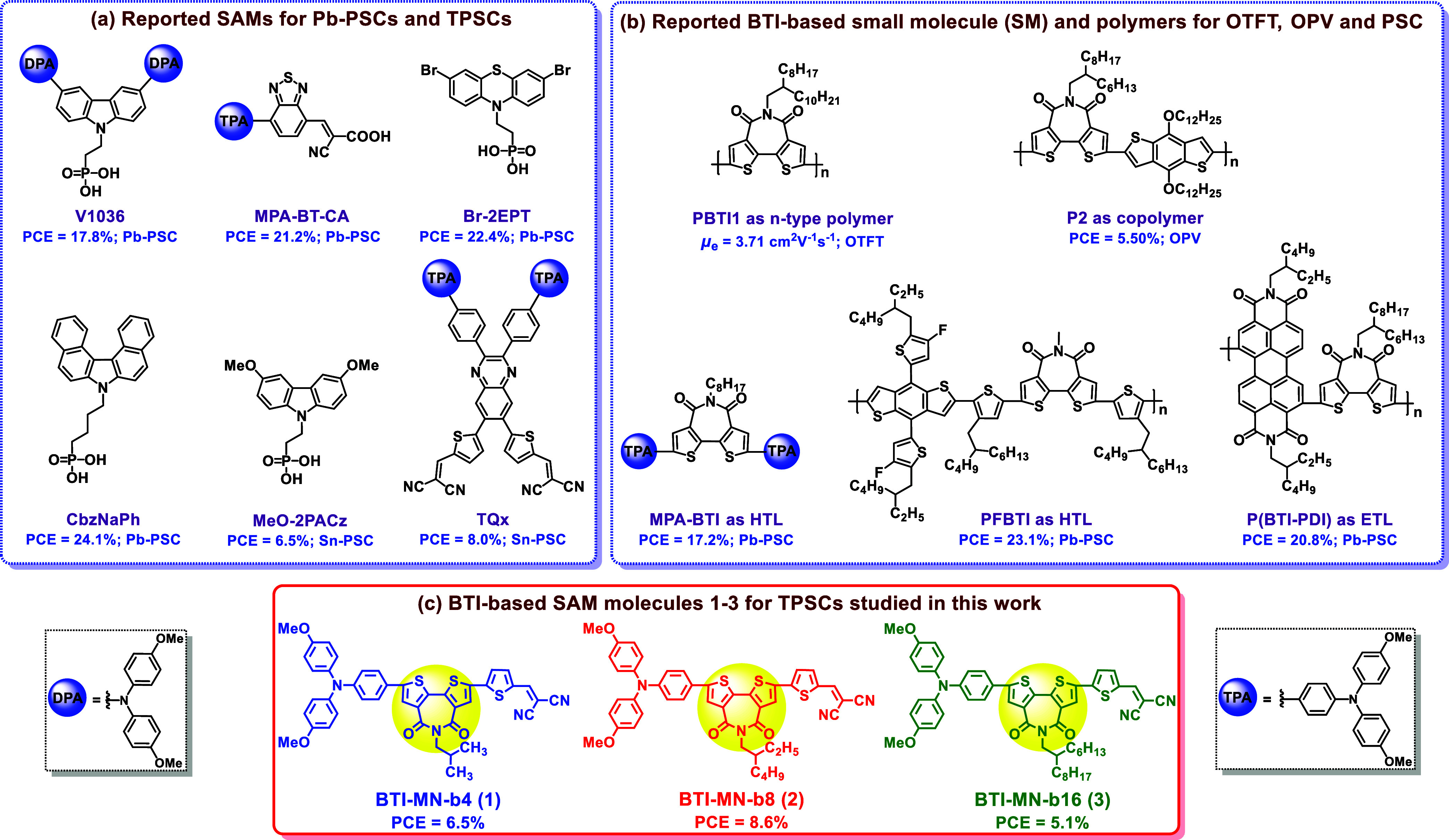
Chemical structures of the (a) reported SAMs for Pb-PSCs and TPSCs; (b) BTI-based small molecule and polymers for OTFTs, OPVs, and PSCs; and (c) BTI-based SAM molecules **1**–**3** for TPSCs studied in this work.

Thus far, several π-conjugated organic molecules have been explored as SAMs in Pb-PSCs, serving as passivants and additives to enhance performance.^38^ For instance, dimethoxydiphenylamine-substituted carbazole **V1036** was first designed by Getautis et al. and used as the dopant-free hole-transporting SAM, achieving a PCE of 17.8%,^39^ as shown in Figure 1a. Guo et al. developed benzo[*c*][1,2,5]-thiadiazole (BT)-based **MPA-BT-CA** as a donor–acceptor-type molecule and applied it in inverted Pb-PSCs, achieving a high PCE of 21.24%.^40^ Similarly, Hong et al. synthesized a phenothiazine-based **Br-2EPT** SAM that minimizes nonradiative recombination losses, leading to an improved PCE of 22.44%.^36^ Furthermore, Jen et al. developed carbazole-derived helical π-expanded **CbzNaph**, which formed a densely packed and ordered monolayer and thus resulted in an excellent PCE of 24.1% with increased stability.^41^

Currently, TPSCs are becoming a promising alternative for advancing lead-free PSCs, achieving record PCEs of 15.7^42^ and 15.38%^43^ by using 4-fluorophenethylamine hydrobromide as an interfacial dipole and isomeric fulleropyrrolidines as additives, respectively. Although TPSCs still lag behind their lead-based counterparts in performance, tin perovskites have a smaller bandgap compared to lead perovskites, suggesting that TPSCs could potentially achieve a greater theoretical PCE than Pb-PSCs.^44,45^ To prevent Sn^2+^/Sn^4+^ oxidation, passivate surface flaws, and control crystal formation in TPSCs, various additive engineering strategies have been employed.^46−56^ As an example, our group recently implemented SAMs of **MeO-2PACz**(57) and **TQx**(58) in TPSCs, achieving PCEs of 6.5 and 8.0%, respectively (Figure 1a).

Bithiophene imide (BTI) is a unique building block for the development of polymeric semiconductors and small molecules for applications of OTFTs,^59−61^ OPVs,^62,63^ and PSCs^64−66^ (Figure 1b). As seen in the figure, BTI is a representative electron-deficient polycyclic organic building block with a planar conjugated backbone, short π–π distance, and adjustable solubilizing alkyl groups. Inspired by these excellent properties, a series of BTI-based SAM molecules were developed in the present study, utilizing BTI as the core. The high-electron donating capability of 4,4′-dimethoxytriphenylamine means that it can be used as an effective electron donor, facilitating efficient charge transport to the perovskite layer. As for the anchoring groups, an electron-withdrawing cyanoacrylic acid or dicyanovinyl group has been reported. Lewis base characteristics of these groups make them efficient passivation sites for lead perovskites, which facilitates interaction with Pb^2+^ ion defects.^67^ Similarly, carboxylic and cyano groups can adhere to the ITO surface, altering its work function and enhancing interfacial interactions.^68^ Furthermore, these highly polar functional groups increase the solubility of specific compounds in polar solvents, which is advantageous for solution-processable device fabrication.^40^ The dicyanovinyl group is thus utilized as the anchoring unit for the development of new **BTI-MN** SAMs for TPSCs.

Figure 1c illustrates the chemical structures of three new SAM molecules developed and studied in this work, named **BTI-MN-b4** (**1**), **BTI-MN-b8** (**2**), and **BTI-MN-b16** (**3**). With the anchoring groups (CN) positioned at one end of the BTI core and the donor moiety 4,4′-dimethoxytriphenylamine at the other end, the new SAMs demonstrate significantly enhanced anchoring strength on the surface of NiOx/ITO, leading to a suitable molecular alignment and improved interaction with the perovskite layer. It is corroborated by the single crystal structure of the **BTI-MN-b8** (**2**) molecule, grown using the slow solvent evaporation method. The presence of intramolecular interactions could facilitate the formation of a uniform and dense SAM produced on the NiOx/ITO substrate, promoting efficient charge transport. Combining these new BTI-based SAMs with NiOx as HTMs for TPSCs, a high power conversion efficiency (PCE) of 8.6% is demonstrated by using **BTI-MN-b8** (**2**). This represents, to the extent of our understanding, the prime example of using a simple dye-based SAM in combination with NiOx as an HTM to achieve a PCE exceeding 8% in high-performance TPSCs. This study aims to showcase the synthesis of organic SAMs (**1**–**3**) of the D–A type for use in TPSCs with NiOx. By reducing trap-assisted recombination, minimizing the energy offset between NiOx and perovskite, improving perovskite crystallization, and enhancing the surface properties of the HTM, we ultimately boost the overall performance of PSCs.

## Results and Discussion

### Synthesis

**Scheme 1:**
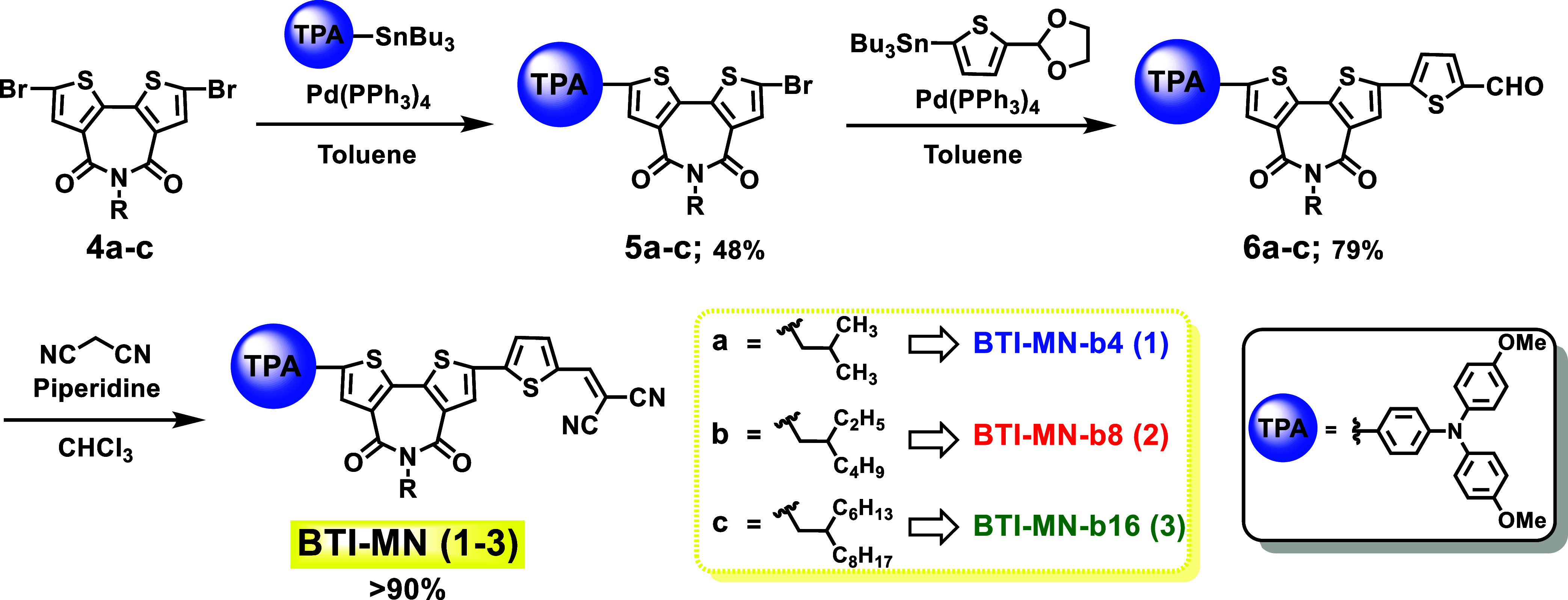
Synthetic Route to BTI-Based SAM Molecules **1**–**3**

The synthetic pathway for BTI-based SAM compounds **1**–**3** is given in Scheme 1. First, dibrominated BTIs **4a**–**c** were prepared using methods previously described in the literature.^59,60^ Further, selective monocoupled compounds **5a**–**c** were synthesized through Stille coupling of **4a**–**c** with stannylated 4,4′-dimethoxytriphenylamine using Pd(PPh_3_)_4_ catalyst. The second Stille coupling reaction of **5a**–**c** was carried out with (5-(1,3-dioxolan-2-yl)thiophen-2-yl)tributylstannane to afford intermediate BTI-aldehydes **6a**–**c**. Furthermore, Knoevenagel condensation of **6a**–**c** with malononitrile in pyridine and refluxing chloroform yields the target **BTI-MN** compounds (**1**–**3**) in good yields (>90%). Methanol recrystallization was used to obtain pure BTI-based SAM molecules **1**–**3**. These compounds exhibit good solubility in chlorobenzene, chloroform, and tetrahydrofuran. Furthermore, structural characterization of all of the target compounds was performed using ^1^H and ^13^C NMR and mass spectrometry (refer to the Supporting Information).

### Physical Characterization

**Figure 2:**
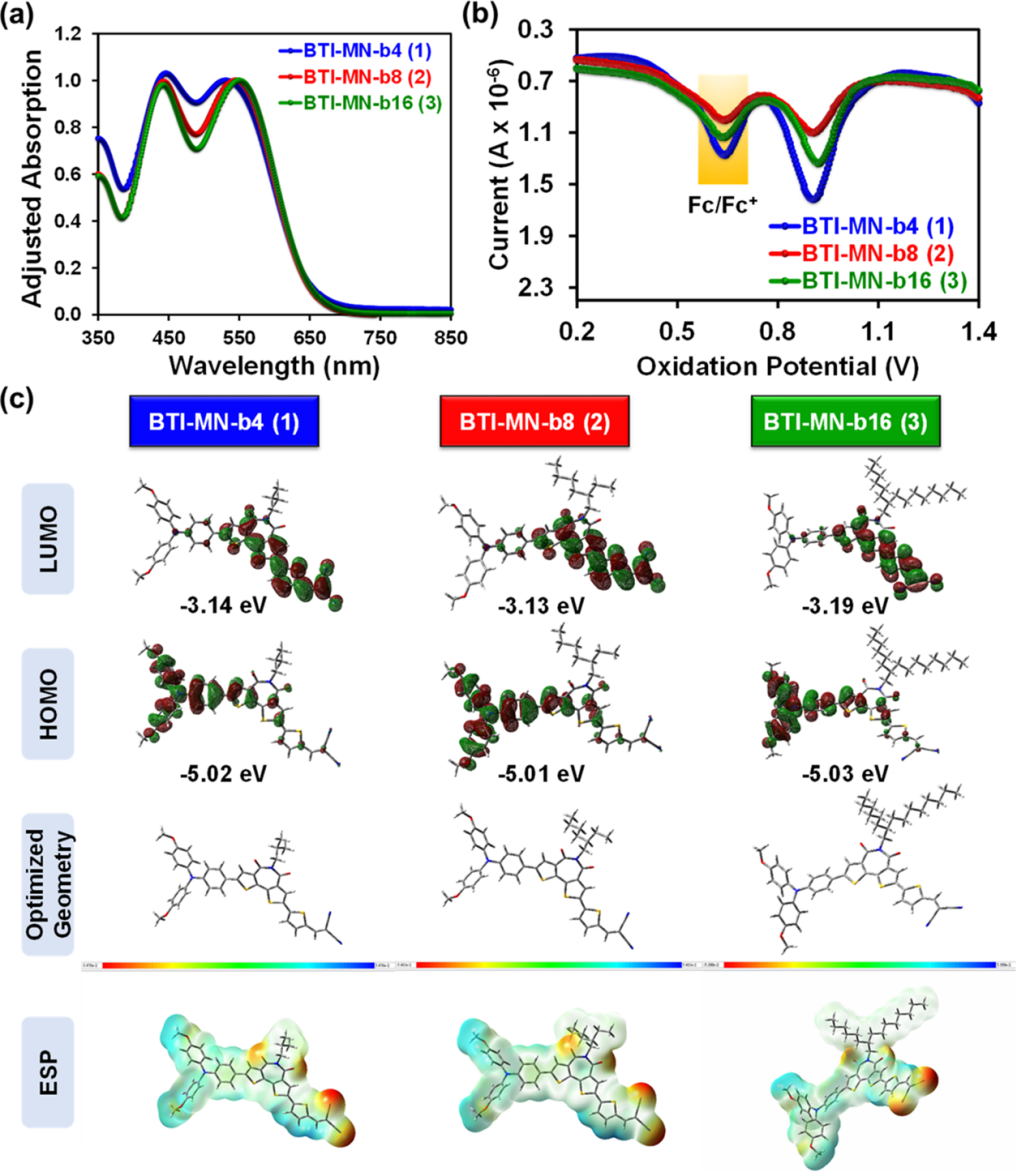
(a) UV–vis absorption spectra; (b) DPV curves; and (c) DFT-derived energy levels and electrostatic surface potential (ESP) mapping of **BTI-MN** compounds.

Table 1 summarizes the physical characteristics of BTI-based SAM molecules **1**–**3**. Thermal analyses of the newly synthesized SAM molecules were performed using thermogravimetric analysis (TGA) (Figure S1). TGA scans revealed that all three compounds possess exceptional thermal stability with ∼5% weight loss temperature at 274, 377, and 416 °C for **1**–**3**, respectively. As shown in Table 1, **BTI-MN-b4** has the highest melting temperature of the series. This is attributed to its shorter side chain, which results in a melting temperature dominated by the rigid backbone. As the length of the branch chains increases from −isobutyl to −ethylhexyl and −hexyldecyl, the melting temperature decreases due to reduced core–core intermolecular interactions as chain mobility increases at higher temperatures.^69^ The optical absorption properties of all **BTI-MN** compounds were measured in *o*-dichlorobenzene (*o*-DCB) solutions. Figure 2a shows the UV–vis absorption spectra, which exhibit nearly identical peak maxima (λ_max_) around ∼540 nm for all three compounds. The optical bandgap, extracted from the absorption onset of the spectra, is approximately 1.83 eV. Consequently, changes in side-chain substituents therefore show similar optical properties and do not significantly affect the π-conjugated backbone. Differential pulse voltammetry (DPV) was performed in *o*-DCB at 25 °C to examine the electrochemical characteristics of SAM molecules **1**–**3** (Figures 2b and S2), using tetrabutylammonium hexafluorophosphate as the electrolyte. Ferrocene served as an internal standard to calibrate the oxidation potentials of SAMs **1**–**3**, with a reference potential set at +0.64 V.^70^ The equation *E*_HOMO_ = −(4.44 + *E*_ox_) was used to calculate the HOMO energy levels of SAM molecules **1**–**3**. The first oxidation and reduction peaks of compounds **1**–**3** are located around +0.91 and −0.74 V, respectively, resulting in *E*_HOMO_ values of −5.35 eV and *E*_LUMO_ values of −3.70 eV for compounds **1**–**3**, respectively. As expected, the electrochemical properties remain largely unaffected by variations in the side-chain substituents of the conjugated backbones, and the energy levels of compounds **1**–**3** are nearly the same.

**Table 1:** Physical Characterization Data of **BTI-MN** SAMs

compound	*T*_d_^a^ [°C]	*T*_m_^b^ [°C]	λ_max_(sol)^c^ [nm]	Δ*E*_g_^d^ [eV]	*E*_ox_^e^ [V]	HOMO^f^ [eV]	*E*_red_^e^ [V]	LUMO^f^ [eV]	Δ*E*_g_^g^ [eV]
**BTI-MN-b4** (**1**)	274	248	531	1.81	0.91	–5.35	–0.74	–3.70	1.65
**BTI-MN-b8** (**2**)	377	191	544	1.85	0.91	–5.35	–0.74	–3.70	1.64
**BTI-MN-b16** (**3**)	416	162	549	1.84	0.92	–5.36	–0.73	–3.71	1.65

a By TGA.

b From the melting point apparatus.

c Determined in *o*-DCB.

d Calculated using 1240/λ_abs_ (onset).

e By DPV in *o*-DCB.

f HOMO/LUMO = −(4.44 + *E*_ox_/*E*_red_) vs NHE.

g Δ*E*_g_ = *E*_LUMO_ – *E*_HOMO_ from DPV.

### Theoretical Calculations

To investigate the electronic structures of BTI-SAMs, DFT calculations were employed at the B3LYP/6-31G* level of theory using the Gaussian 09W program (Figure 2c). The HOMOs of SAM molecules are predominantly found on the triphenylamine units, whereas the LUMOs are observed on the bithiophene imide backbone and the thiophene spacers connected to the anchoring units.^58^ DFT-derived *E*_HOMO_ and *E*_LUMO_ of compounds **1**–**3** are around −5.02 and −3.15 eV, respectively. It is noteworthy that the energy gaps determined using computational, optical, and electrochemical methods are nearly comparable, indicating the consistency between the values. In addition, electrostatic potential (ESP) surface analysis of the **BTI-MN** SAMs was performed to analyze the molecular charge distribution and examine the interaction between the NiOx/ITO surface and the anchoring group sites (Figure 2c). As a result, DFT calculations and ESP mapping images of the three BTI-based SAMs indicate that a high density of negative charges is predominantly found on the cyano anchoring groups.^71^ This facilitates the formation of SAMs on the NiOx/ITO surface through strong interactions with the anchoring groups,^72^ potentially enhancing charge transport at the SAM/NiOx interface in inverted TPSCs.

### Single Crystal Structure

**Figure 3:**
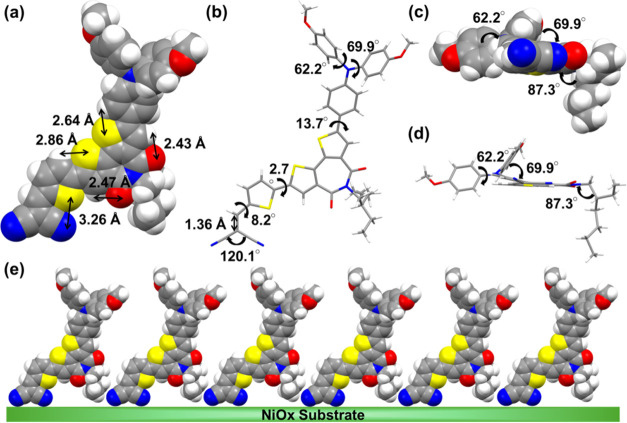
Single crystal structure of compound **2**. (a) Top view of **BTI-MN-b8** molecule with intramolecular interactions in a space-filling model; (b) top view of **BTI-MN-b8** molecule with various twisted angles in stick model; (c, d) front views of **BTI-MN-b8** molecule the space-filling and stick models, respectively; and (e) expected packing pattern of SAM on NiOx/ITO substrate (front view).

In order to obatin further insight into the structure of the SAM molecule, a single crystal of **BTI-MN-b8** (**2**) was grown, and the diffraction-derived single crystal structure of **BTI-MN-b8** (**2**) molecule is presented in Figures 3, S3, and S4; the respective crystal data are given in Table S1. These results showed that the **BTI-MN-b8** molecule crystallizes in the orthorhombic system with the Pca2_1_ space group. Figure 3a illustrates intramolecular distances of 3.26, 2.43–2.47, and 2.64–2.86 Å for N···S, O···H, and S···H, respectively, while the SAM molecule stands upright with two CN groups serving as the anchoring points. These interactions could facilitate the formation of highly ordered SAMs on the substrate with a dense and tilted texture. As shown in Figure 3b, the short double bond characteristic of 1.36 Å for C=C between thiophene and MN groups, along with the relatively small twisted angles of the thiophene spacer to MN (8.2°) and BTI core (2.7°), confirms the good π-conjugation between the central BTI core and the two end-anchoring CNs, which facilitates efficient charge transport. Furthermore, the presence of more twisted angles (62.2 and 69.9°) measured for the phenyl rings on the TPA group enhances solubility, enabling facile device fabrication via solution processing, thus yielding high-quality films for attaining a high PCE. In addition, the wide angle (120.1°) between the two CN anchoring groups should benefit the SAM molecule in standing firmly on the NiOx substrate. Figure 3c,d showcases the front views of the **BTI-MN-b8** molecule with obvious twisted angles of phenyl rings and the alkyl chain situated perpendicularly to the central BTI core (87.3°). The expected packing pattern of **BTI-MN-b8** SAM molecules standing on the NiOx substrate is demonstrated either with two legs (two CN) or with one leg (one CN) (Figures 3e and S4), promoting charge transfer efficiency for high-performance TPSCs. Notably, the side chain (b8) herein plays a key role in the packing of SAM on the NiOx film. For the bidentate bonding (Figure 3e), the side chain interacts with the two anchoring groups (CN), while for the monodentate bonding (Figure 3e), the side chain interacts with the central **BTI** core. Therefore, the size of the alkyl chain would affect the SAM packing on the NiOx surface, for which the b8 side chain has an appropriate size to avoid dye aggregation and packing hindrance for the SAM to form a uniform layer on NiOx. In contrast, for the b4 side chain (**BTI-MN-b4**), the size is too small so that dye aggregation may occur on the NiOx surface. For the case of the b16 side chain (**BTI-MN-b16**), the size is too large, so packing hindrance may occur to affect the uniformity of the SAM on the NiOx surface.

### Thin-Film Characterizations

**Figure 4:**
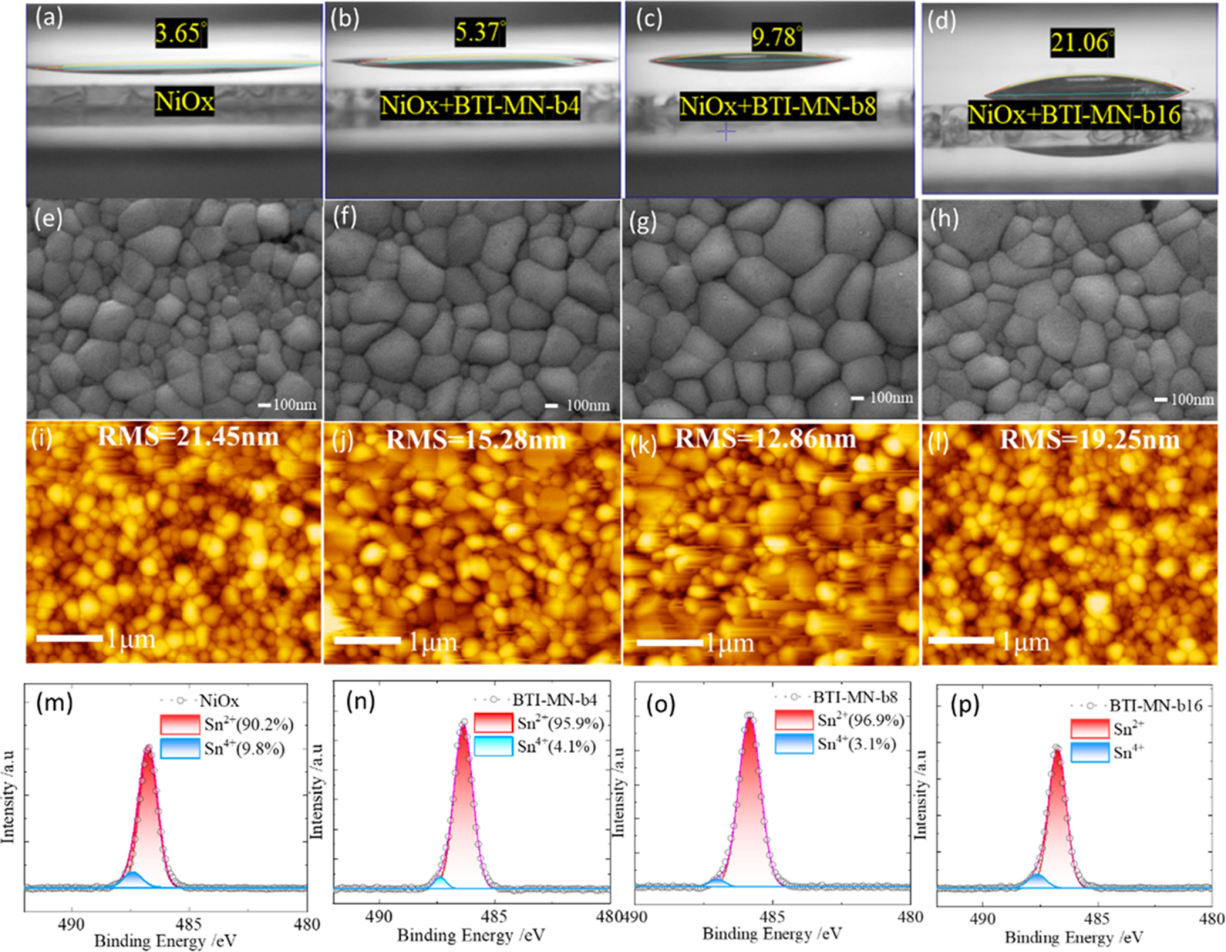
(a–d) Contact angle of SnI_2_ precursor on NiOx and NiOx/SAM surfaces. (e–h) SEM, (i–l) AFM, and (m–p) XPS of tin perovskites made on NiOx and NiOx/SAM surfaces.

We expect that the presence of NiOx induces the oxidation of tin perovskite mainly at the interface between the perovskite and NiOx layers, thereby forming a barrier that hinders hole extraction. Figure 4 shows the wettability and crystal morphology of tin perovskites deposited on bare NiOx and NiOx treated with various SAMs **1**–**3**. Initially, NiOx/SAM layers were applied to ITO substrates via a dipping process. NiOx aims to improve the surface hydrophilicity. The contact angles measured for the SnI_2_ precursor solution on SAM films were recorded as 9.56° for **BTI-MN-b4**, 13.02° for **BTI-MN-b8**, and 27.22° for **BTI-MN-b16**, as presented in Figure S5a–c. Functionalization of NiOx with SAMs significantly enhanced hydrophilicity, reducing the contact angles for **BTI-MN-b4** to 5.37°, **BTI-MN-b8** to 9.78°, and **BTI-MN-b16** to 21.06°, while the contact angle for the untreated NiOx film was slightly lower at 3.65°, as detailed in Figure 4a–d. This modification of the surface notably supports the further deposition of FAI, forming FASnI_3_ perovskites with a favorable morphology. The increase in hydrophilicity indicates a link between the processability of SAMs and their performance in devices. It is evident that only SAMs with optimal processability result in enhanced device performance. Among the three SAMs, NiOx/**BTI-MN-b4** and NiOx/**BTI-MN-b8** demonstrated lower contact angles compared to NiOx/**BTI-MN-b16**.

Scanning electron microscopy (SEM) and atomic force microscopy (AFM) were used to analyze the morphologies of tin perovskite films applied to various NiOx/SAMs. Figure 4e–h shows the top-view SEM images of the perovskite films on different NiOx/SAMs, with the films of **BTI-MN-b8** (Figure 4g) featuring larger and more uniform crystals compared to the others, a finding supported by the AFM results shown in Figure 4i–l. These AFM images were examined to assess the surface roughness of the tin perovskites on different NiOx/SAMs, for which those with the **BTI-MN-b8**-based perovskites show smaller surface roughness than the others. Figure S6 presents AFM images of the SAMs applied to ITO (a–c) and NiOx/ITO (d–f) substrates. It can be seen that the surface of bare ITO is very rough, so that the SAM molecules were randomly distributed on the ITO surface (Figure S6a–c). After deposition of NiOx on the ITO substrate, the roughness of the ITO surface was modified by the NiOx layer so that the corresponding AFM images exhibit a flat feature, as clearly seen in Figure S6d–f. We therefore highlight here the role of the NiOx layer not only to modify the hydrophilicity of the film but also to fill the hollow surface of the ITO substrate. As a result, the molecules **1**–**3** are feasible to be deposited on a flat NiOx-modified ITO surface to form a self-assembled monolayer, as schematically demonstrated in Figures 3e and S4b.

Figure S7 presents KPFM images of the SAMs applied to ITO (a–c) and NiOx/ITO (d–f) substrates. The surface potentials of ITO with SAMs **1**–**3** are 2.89, 3.37, and 2.98 mV, respectively, while those for NiOx/ITO with SAMs **1**–**3** are 3.11, 3.52, and 2.99 mV, respectively, for which the **BTI-MN-b8**-based perovskites showed larger surface potential than the others. Furthermore, the SAM-treated ITO substrates exhibited a sheet-like structure, while those treated with NiOx/ITO showed a compact spherical shape. This structural difference enhances the specific surface area in the latter, facilitating the formation of a compact and uniform layer between NiOx/ITO and the perovskite layers. Figure 4m–p shows the XPS Sn^2+^/Sn^4+^ results of the NiOx and varied SAM/NiOx films, as indicated (Table S2). Functionalization of SAM on NiOx significantly reduces the Sn^2+^/Sn^4+^ oxidation, with the largest Sn^2+^/Sn^4+^ ratio occurring on the **BTI-MN-b8** sample (Sn^2+^: 96.9%). The above analysis indicates that the side chains in SAM **1**–**3** do play an important role in the subsequent growth of the perovskite layer, for which the intermediate size of the alkyl chain (**BTI-MN-b8**) gives the best quality of perovskite film.

### Device Performance and Characterizations

**Figure 5:**
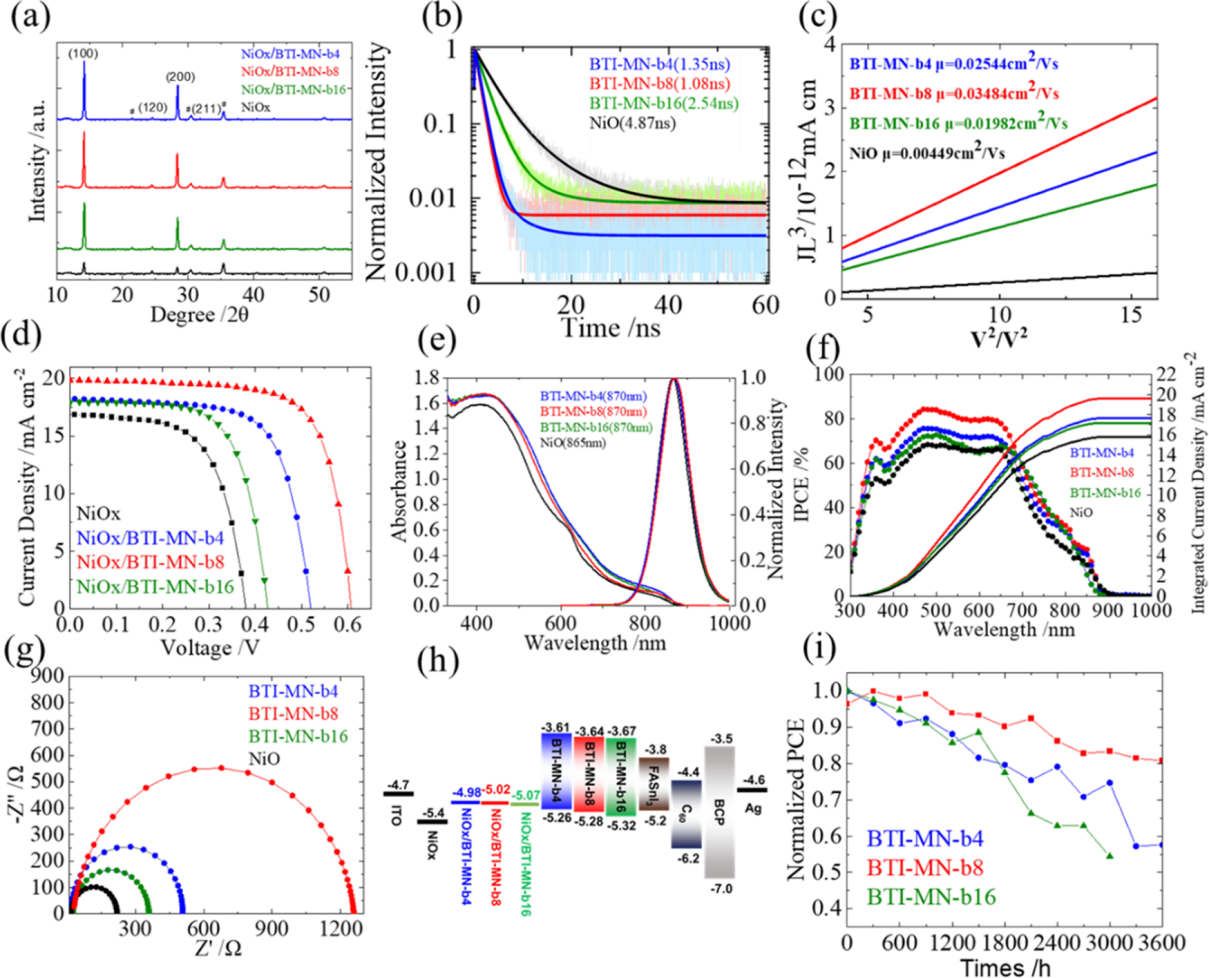
(a) XRD patterns of tin perovskites on NiOx with various SAMs, as specified; (b) PL decay profiles obtained using TCSPC; (c) SCLC measurements of tin perovskites on NiOx with the various indicated SAMs; (d) *J*–*V* characteristic curves; (e) IPCE and (f) UV–vis absorption and PL spectroscopy of tin perovskites on NiOx with various SAMs, as specified; (g) EIS Nyquist plots for devices composed of tin perovskites deposited on NiOx with various SAMs, as specified; (h) energy-level diagram; and (i) long-term stability of devices containing NiOx with various SAMs, as specified. The highest-performing device, NiOx/**BTI-MN-b8**, achieved a PCE of 8.6%.

The X-ray diffraction (XRD) patterns of tin perovskites (Figure 5a) show a higher intensity for crystallization when the perovskite is deposited on these SAMs rather than on NiOx. NiOx, when used as a distinct hole transport layer, tends to oxidize SnI_2_, which decreases the crystallinity of tin perovskite, as shown in the (100) facet. As a result, the crystallization intensity observed in XRD of the bare NiOx film is significantly reduced. SAMs mitigate the oxygen defects in NiOx so as to enhance the crystallinity of the perovskite. These results suggest that the SAMs interact with the perovskite through electron donation, effectively preventing oxidation and defect formation at the SAM/perovskite interface while preserving the superior bulk structure and optical properties of the perovskite. A high-resolution grazing incidence wide-angle X-ray scattering (GIWAXS) image (Figure S8) of the NiOx/**BTI-MN-b8** film demonstrates the perovskites’ arrangement and enhanced crystallinity, which can improve the carrier transport and the performance of the NiOx/**BTI-MN-b8** device.

The hole-extraction capabilities of tin perovskite films on various SAMs were assessed using the time-correlated single-photon counting (TCSPC) technique, with excitation at 635 nm from the glass side, and the corresponding photoluminescence (PL) decay profiles are displayed in Figure 5b. These decay profiles were analyzed using a biexponential function model, and the corresponding results are summarized in Table S3. The results show that the hole-extraction ability has a trend with NiOx/**BTI-MN-b8** (1.1 ns) > NiOx/**BTI-MN-b4** (1.4 ns) > NiOx/**BTI-MN-b16** (2.5 ns) > NiOx (4.9 ns). Additionally, space-charge limited current (SCLC) measurements of hole mobilities in the NiOx/SAM thin films indicate that NiOx/**BTI-MN-b8** possesses the highest hole mobility (Figure 5c) of all of the samples, showing its outstanding performance compared to the other SAMs.

Employing a two-step fabrication method, TPSC devices were fabricated with a layered structure with the configuration ITO/NiOx/SAM/FASnI_3_/C60/BCP/Ag. The current–voltage (*J–V*) characteristics shown in Figure 5d indicate that the performances of the NiOx/SAM devices have the following trend: **BTI-MN-b8** (8.6%) leads, followed by **BTI-MN-b4** (6.5%), **BTI-MN-b16** (5.1%), and NiOx-only (4.0%), with the corresponding photovoltaic parameters listed in Table S4. This performance hierarchy aligns with the morphological and passivation features noted in Figure 4, where devices incorporating NiOx with **BTI-MN-b4** and **BTI-MN-b16** exhibited lower performance due to inferior morphology, as demonstrated in the SEM (Figure 4e–h) and AFM (Figure 4i–l) images. The exceptional efficiency (PCE = 8.6%) of the NiOx/**BTI-MN-b8** device is attributed to its great film morphology (Figure 4c,g,k), rapid hole extraction (Figure 5b), and enhanced hole mobility (Figure 5c). The UV–vis absorption and PL spectra of these SAM molecules are shown in Figure 5e, which do not show any discrepancies for each molecule. The incident photon-to-current conversion efficiency (IPCE) spectra for the NiOx/SAM devices, presented in Figure 5f, along with the integrated short-circuit current densities (*J*_SC_), reinforce the results from the *J*–*V* curves (Figure 5d). These findings particularly highlight the excellent *J*_SC_ value of the NiOx/**BTI-MN-b8** device, which is consistent with its IPCE spectral response shown in Figure 5f, particularly within the 350–700 nm spectral range.

Electrochemical impedance spectral (EIS) measurements were obtained to analyze the charge recombination characteristics of the NiOx/SAM TPSCs in comparison to that of the NiOx-only device, with measurements performed in the dark at a 0.5 V bias. As depicted in Figure 5g, the Nyquist plots for all devices are a single semicircle, which was interpreted using a simple RC equivalent circuit model to fit the EIS data. The fitted impedances provide insight into the extent of charge recombination. The order of charge recombination resistances has the trend NiOx/**BTI-MN-b8** > NiOx/**BTI-MN-b4** > NiOx/**BTI-MN-b16** > NiOx-only, which is consistent with the trend of *V*_OC_ showing the same order. This sequence thus highlights the superior performance of the NiOx/**BTI-MN-b8** device, with the largest *V*_OC_ value of all of the devices.

The techniques of ultraviolet photoelectron spectroscopy (UPS) and UV–vis spectroscopy were employed to determine the HOMO and LUMO energy levels of the SAM and NiOx/SAM films. The UPS raw data for samples with and without NOx are displayed in Figures S9 and S10, respectively. Figure 5h presents the energy-level diagram for all SAMs, with and without NOx modification. The diagrams reveal that SAMs modified with NiOx exhibit higher HOMO levels compared to those without NiOx, with the energy level of the NiOx/**BTI-MN-b8** film closely aligning with that of FASnI_3_, supporting the performance results shown in Figure 5d.

Figure 5i illustrates the shelf-storage performance stability of all NiOx/SAM TPSCs, showing a steady decline in performance over 3600 h. Among them, the NiOx/**BTI-MN-b8** device exhibited outstanding long-term stability, maintaining approximately 80% of its initial efficiency after 3600 h. This exceptional durability of the NiOx/**BTI-MN-b8** device can be explained by its superior optoelectronic properties, morphology, crystallinity, and mobility, as previously discussed. The reproducibility of these findings was verified through performance statistics from 20 devices for each SAM, with the corresponding photovoltaic parameters summarized in Tables S5–S8 and depicted in box plot form in Figure S11. Additionally, the NiOx/**BTI-MN-b8** device showed no effect of hysteresis, as evidenced by the *J*–*V* scan curves shown in Figure S12. Moreover, under one sun illumination in ambient air conditions, the performance at the maximum power point (MPP) of the unencapsulated NiOx/**BTI-MN-b8** device remained over 80% for 5 h (Figure S13), further confirming its superior stability under light soaking conditions. Therefore, this study demonstrates that the NiOx interlayer plays a crucial role in enhancing SAM functionality, promoting the growth of a large, uniform perovskite layer to enhance the device performance for this series of SAM devices. For the three SAM molecules with varied alkyl side chains, our results indicate that the one with medium side-chain length (**BTI-MN-b8**) could help in growing a perovskite layer with a better crystal morphology, smaller film roughness, better crystallinity, retarded charge recombination, greater hole mobility, and better hole-extraction ability than the others. As a result, the **BTI-MN-b8** device showed excellent device performance and stability compared with all of the other devices.

## Conclusions

Three bithiophene imide-based molecules, designated as **BTI-MN-b4** (**1**), **BTI-MN-b8** (**2**), and **BTI-MN-b16** (**3**), were synthesized to serve as SAMs for the TPSCs. DFT calculations confirmed that the triphenylamine groups were attached to one side of the BTI core, facilitating effective charge transport to the perovskite layer, while cyano groups, conjugated with the thiophene unit, were attached to another side of the BTI core, serving as anchoring groups for strong interaction with the NiOx/ITO surface; NiOx plays an important role in modifying the hydrophilicity and surface roughness of the ITO substrate for molecules **1**–**3** to form uniform SAMs on it. Additionally, solubilizing alkyl groups attached to the BTI core were expected to mitigate dye aggregation and control the SAM packing on the NiOx/ITO substrates. Moreover, the single crystal structure of the **BTI-MN-b8** (**2**) molecule exhibits intramolecular interactions, improved π-conjugation, and appropriate dihedral angles could facilitate the formation of a uniform and dense SAM produced on the NiOx/ITO substrate, promoting efficient charge transport. We found that dicyanovinyl was an effective anchoring group for all of the three organic molecules to form SAMs on the NiOx/ITO surface, resulting in the formation of smooth and uniform tin perovskite nanocrystals with excellent morphology and crystallinity. When only NiOx was used as an HTM in a TPSC, the devices exhibited a poor PCE of 4.0%. To enhance efficiency, we employed a novel approach that combines new organic SAMs (**1**–**3**) with NiOx as the HTMs. This led to a remarkable PCE of 8.6% for a SAM device based on NiOx/**BTI-MN-b8**; the SAM devices based on NiOx/**BTI-MN-b4** and NiOx/**BTI-MN-b16** exhibited PCEs of 6.5 and 5.1%, respectively. The outstanding efficiency of the NiOx/**BTI-MN-b8** devices can be attributed to their favorable film morphology, rapid hole extraction, enhanced hole mobility, and retarded charge recombination. Furthermore, we performed various characterizations, including contact angle measurements, SEM, AFM, XPS, XRD, GIWAXS, TCSPC, UPS, SCLC, and EIS, to comprehensively understand the optoelectronic and photovoltaic properties of the NiOx/SAM/perovskite thin films and the corresponding device performances. Our work thus presents a methodology for combining new organic SAM molecules with NiOx HTM in TPSCs to achieve excellent performance and enhanced stability.

## Experimental Section

### Materials

Starting materials (from Sigma-Aldrich, Alfa, or TCI Chemical Co.) were reagent grade and were used without further purification unless otherwise indicated. Reaction solvents (toluene and chloroform) were distilled under nitrogen from sodium/benzophenone ketyl, and halogenated solvents were distilled from CaH_2_. Compounds 2,8-dibromo-5-alkyl-4*H*-dithieno[3,2-*c*:2′,3′-*e*]azepine-4,6(5*H*)-dione (**4a**–**c**) and stannylated 4,4′-dimethoxytriphenylamine were synthesized as reported previously.^59,60^

### General Procedures for Final Target Compounds (**1**–**3**)

Under anhydrous conditions, compounds **6a**–**c** (1 equiv) and malononitrile (20 equiv) were dissolved in 20 mL of CHCl_3_. To the resulting solution, pyridine (1 mL) was added slowly and then heated to reflux for 24 h. The reaction mixture was cooled to room temperature and extracted with dichloromethane. The organic layer was dried over Na_2_SO_4_, concentrated, and recrystallized from methanol to give target **BTI-MN** compounds (**1**–**3**) as a deep red solid (yield >90%).

#### Synthesis of **BTI-MN-b4** (**1**)

^1^H NMR (500 MHz, CDCl_3_): δ (ppm) 8.01 (s, 1H), 7.80–7.77 (m, 2H), 7.69 (m, 1H), 7.40 (d, *J* = 8 Hz, 2H), 7.34 (m, 1H), 7.10 (d, *J* = 7 Hz, 4H), 6.90–6.86 (m, 6H), 4.17 (d, *J* = 7.6 Hz, 2H), 3.81 (s, 6H), 2.19–2.14 (m, 1H), 0.96–0.95 (m, 6H). ^13^C NMR (125 MHz, CDCl_3_): δ 161.72, 161.42, 156.67, 149.93, 149.70, 146.32, 144.93, 139.83, 139.44, 138.78, 135.05, 134.75, 133.13, 133.03, 132.33, 132.20, 127.33, 126.71, 125.70, 123.07, 119.19, 114.96, 113.82, 113.06, 77.86, 55.51, 52.24, 29.68, 27.34, 20.38. HRMS (MALDI, [M]^+^) calcd. for C_42_H_32_N_4_O_4_S_3_: 752.1586, found: 752.1580.

#### Synthesis of **BTI-MN-b8** (**2**)

^1^H NMR (500 MHz, CDCl_3_): δ (ppm) 8.01 (s, 1H), 7.80–7.76 (m, 2H), 7.68 (d, *J* = 4 Hz, 1H), 7.40 (m, 2H), 7.32 (d, *J* = 4 Hz, 1H), 7.10 (m, 4H), 6.88 (m, 6H), 4.29–4.19 (m, 2H), 3.82 (s, 6H), 1.86–1.83 (m, 1H), 1.36–1.25 (m, 8H), 0.92–0.86 (m, 6H). ^13^C NMR (125 MHz, CDCl_3_): δ 161.71, 161.37, 156.68, 149.93, 149.64, 146.30, 144.92, 139.80, 139.45, 138.74, 135.06, 134.73, 133.11, 132.95, 132.33, 132.16, 127.36, 126.69, 125.64, 123.013, 119.11, 114.96, 113.81, 113.06, 77.84, 55.51, 49.26, 37.878, 30.83, 28.70, 24.13, 23.12, 14.09, 10.71. HRMS (MALDI, [M]^+^) calcd. for C_46_H_40_N_4_O_4_S_3_: 808.2212, found: 808.2206.

#### Synthesis of **BTI-MN-b16** (**3**)

^1^H NMR (500 MHz, CDCl_3_): δ (ppm) 8.00 (s, 1H), 7.79–7.76 (m, 2H), 7.68 (d, *J* = 4 Hz, 1H), 7.39 (m, 2H), 7.32 (d, *J* = 4 Hz, 1H), 7.10 (m, 4H), 6.88–6.86 (m, 6H), 4.23 (d, *J* = 7.5 Hz, 2H), 3.81 (s, 6H), 1.90 (m, 1H), 1.23 (m, 24H), 0.85–0.83 (m, 6H). ^13^C NMR (125 MHz, CDCl_3_): δ 161.72, 161.36, 156.68, 149.93, 149.63, 146.31, 144.93, 139.81, 139.41, 138.72, 135.09, 134.73, 133.15, 132.92, 132.32, 132.16, 127.35, 126.69, 125.64, 123.04, 119.14, 114.96, 113.81, 113.05, 77.86, 55.50, 49.63, 36.50, 31.90, 31.83, 31.78, 30.08, 29.76, 29.57, 29.32, 26.51, 26.48, 22.66, 14.08. HRMS (MALDI, [M]^+^) calcd. for C_46_H_40_N_4_O_4_S_3_: 920.3464, found: 920.3458.

## Supporting Information Available

Experimental details, TGA curves, DPV curves, single crystal data, morphological analysis, UPS, box plots, hysteresis, MPPT, tables, ^1^H NMR, ^13^C NMR, and HRMS spectra. (PDF)
