# Supplementary material for: Bithiophene Imide-Based Self-Assembled Monolayers (SAMs) on NiOx for High-Performance Tin Perovskite Solar Cells Fabricated Using a Two-Step Approach
Source: ACS Appl Mater Interfaces. 2024 Dec 27;17(1):952–62. doi: 10.1021/acsami.4c15688 (PMC11783363; doi:10.1021/acsami.4c15688)
Supplement: Supplementary file 1 — am4c15688_si_001.pdf [file am4c15688_si_001.pdf]

Supporting Information

Bithiophene Imide-based Self-Assembled Monolayers (SAMs) on NiOx for High-Performance Tin Perovskite Solar Cells Fabricated Using a Two-Step Approach

Arulmozhi Velusamy, [a, ‡] *Chun-Hsiao Kuan,*[b, ‡] *Tsung-Chun Lin,* [b] *Yun-Sheng Shih,* [b] *Cheng-Liang Liu,* [c] *De-You Zeng,*[a] *Yu-Gi Li,*[a] *Yu-Hao Wang,* [c] *Xianyuan Jiang,* [d] *Ming-Chou Chen,**[a] *and Eric Wei-Guang Diau**[b, e]

[a] Department of Chemistry and Research Center of New Generation Light Driven Photovoltaic Modules, National Central University, Taoyuan 32001, Taiwan.

[b] Department of Applied Chemistry and Institute of Molecular Science, National Yang Ming Chiao Tung University, 1001 Ta-Hseuh Rd., Hsinchu 300093, Taiwan.

[c] Department of Materials Science and Engineering, National Taiwan University, Taipei 10617, Taiwan.

[d] School of Physical Science and Technology, ShanghaiTech University, Shanghai 201210, China.

[e] Center for Emergent Functional Matter Science, National Yang Ming Chiao Tung University, 1001 Ta-Hseuh Rd., Hsinchu 300093, Taiwan.

*Corresponding authors' email addresses:
Email: mcchen@ncu.edu.tw

Email: diau@nycu.edu.tw

Experimental Section

Chemicals:

NiOx nanoparticles (Lumtech), FAI (Greatcell Solar), SnI_2 (99.999%, Alfa Aesar), SnF_2 (99%, Aldrich), DMSO and DMF (Aldrich), chlorobenzene (Aldrich), IPA (Aldrich), hexafluoro-2-propanol (HFP, TCI), $EDAI_2$ (Greatcell Solar) were used without further purification. $EDAI_2$ was synthesized on reacting excess HI (57%, Alfa Aesar) with 1,2-diaminoethane (99%, Alfa Aesar) in an ice bath, after which the resulting white powder was collected, thoroughly washed with diethyl ether, and stored under 50 ^{o}C vacuum oven for a day. Dry ethanol prepared by distillation was used to make SAM solutions.

Device fabrication:

Commercial ITO glasses, sized 1.9×1.9 cm^2, underwent a cleaning process using IPA, acetone, and distilled water while being sonicated for 30 minutes prior to use. NiOx nanoparticle solution (5mg in IPA:DI water=3:1) was spin-coated at 5000/30nm, elf-assembled monolayer (SAM) molecules were applied using either spin-coating (at 3000 rpm for 30 seconds following a ~5-second rest) or dipping for ~1 day. Solutions of SAM in ethanol, at concentrations of 1.0 mM for spin-coating and 0.2 mM for dipping, were prepared, and the as prepared NiOx-substrates were sonicated for ~1 hour to detach any non-adhered SAM molecules from the NiOx surface. For spin-coating, 50 μL of the solution was used per ITO glass, while for dipping, 20 mL of the solution accommodated six ITO glasses. All procedures, except sonication, were conducted in a nitrogen-filled glove box. A UV-ozone treatment for 30 minutes was carried out before the deposition of SAM. The formulation for the two-step tin perovskite included one solution with SnI2 (0.8 mM), SnF2 (0.16 mM), and EDAI2 (0.008 mM) in DMSO, and another with FAI (20 mg) in an IPA/HFP/CB cosolvent (5:5:2, v/v/v, 1.2 mL). Initially, 40 μL of the SnI2 solution

was spin-coated onto the SAM-modified ITO glasses at 6000 rpm for 1 minute. Then, without prior annealing, 100 μL of the FAI solution was applied for ~40 seconds on the SnI_2-coated substrates, followed by a spin at 5000 rpm for 12 seconds, before being annealed at 70°C for 10 minutes. Finally, through thermal evaporation at approximately 5×10^-6 Torr, an electron transport layer of C60 (~30 nm thick), a hole-blocking layer of BCP (~5 nm thick), and a top metal layer of Ag (~100 nm thick) were deposited.

Characterization of films and devices:

J-V characteristic curves were obtained at a scan rate of 0.07 V/s using a solar simulator (model XES-40S1 by SAN-EI) under standard one sun illumination (AM 1.5G spectrum, 100 mW/cm^2), with calibration performed against a standard silicon reference cell (Oriel, model PN 91150V, VLSI standard). The active area of the devices, defined by a metal mask, was 0.0225 cm^2. Incident photon-to-current efficiency (IPCE) spectra were measured following calibration with a standard silicon photodiode (model S1337-1012BQ by Hamamatsu). X-ray diffraction (XRD) patterns were collected using a Bruker D8-Advance diffractometer equipped with Cu Kα radiation. The morphology and structure of the samples were examined using a field-emission scanning electron microscope (SEM, model SU8010 by Hitachi) and an atomic force microscope (AFM, model VT SPM by SII Nanotechnology Inc.). UV-vis/NIR absorption spectra were captured with a Jasco V-570 spectrophotometer, incorporating a spherical accessory for enhanced sensitivity. Photoluminescence (PL) decay profiles were analyzed using a time-correlated single-photon counting system (TCSPC, Fluotime 200 by PicoQuant), excited by a picosecond pulsed-diode laser (model LDH-635 by PicoQuant, FWHM ~70 ps) at a repetition rate of 8 MHz. Electrochemical impedance spectroscopy (EIS) measurements were carried out in the dark with an AC amplitude of 10 mV across a frequency range of 1 MHz to

1 Hz, applying a variable DC bias (~0.2–0.5 V). PL experiments were performed at an excitation wavelength of 450 nm, with emission spectra monitored in the range of 700 – 1100 nm, using a long-pass filter (650 nm) to block scattered excitation light at the emission monochromator's entrance. X-ray photoelectron spectra (XPS) and ultraviolet photoelectron spectra (UPS) were also acquired using a synchrotron source at different excitation energies at beamline 24A1 of the Taiwan Light Source in the National Synchrotron Radiation Research Center (NSRRC).

Characterization:

^1H and ^{13}C NMR spectra were recorded using a Bruker 500 or a 300 instrument, with reference to solvent signals. Differential scanning calorimetry (DSC) was carried out on a Mettler DSC 822 instrument at a scan rate of 10 K/min. Thermo gravimetric analysis (TGA) was performed on a Perkin Elmer TGA-7 thermal analysis system using dry nitrogen as a carrier gas at a flow rate of 40 mL/min. The UV–vis spectrum was characterized with a JASCO V-670 UV–vis spectrophotometer. Differential pulse voltammetry experiments were performed with a conventional three-electrode configuration (a platinum disk working electrode, an auxiliary platinum wire electrode, and a non-aqueous Ag reference electrode, with a supporting electrolyte of 0.1 M tetrabutylammonium hexafluorophosphate (dry $TBAPF_6$) in the specified dry solvent, using a CHI621C Electrochemical Analyzer (CH Instruments). Under N_2, the anhydrous OSC material was dissolved in above 0.1 M o-$C_6H_4Cl_2$ solution to prepare a 10^{-3} M test solution. In each DPV experiment, 5 mL of the test solution is scanned together with Fc/Fc^+ (also 10^{-3} M; as internal standard) under N_2. electrochemical potentials were referenced to an Fc/Fc^+ internal standard (at +0.64 V). Mass spectrometric data were obtained with an ATS-00670 HRMS instrument.

Synthesis of intermediates

General procedure for the synthesis of 2-(4-(bis(4-methoxyphenyl)amino)phenyl)-8-bromo-5-alkyl-4*H*-dithieno[3,2-*c*:2',3'-*e*]azepine-4,6(5*H*)-dione (**5a-c**): Under anhydrous condition, $Pd(PPh_3)_4$ (0.05 equiv.) was added to a solution of 2,8-dibromo-5-alkyl-4*H*-dithieno[3,2-*c*:2',3'-e]azepine-4,6(5*H*)-dione (**4a-c**; 1 equiv.) and stannylated 4,4'-dimethoxy triphenylamine (1 equiv.) in dry toluene. The resulting mixture was refluxed for 24 hours under nitrogen. After cooling to room temperature, the solvent was evaporated and the obtained residue was purified by column chromatography with ethyl acetate/hexanes as the eluent.

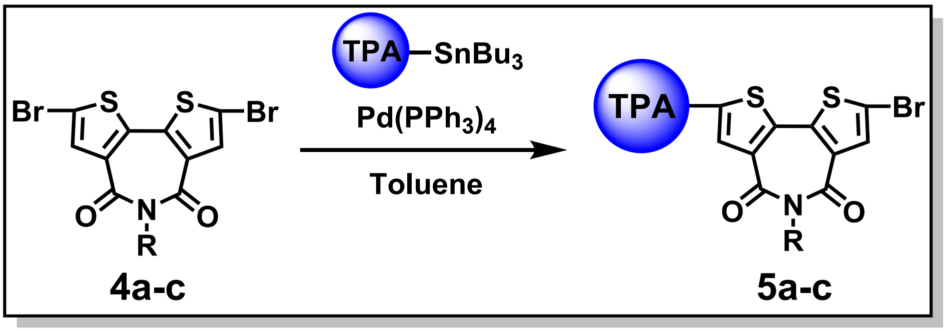

Scheme S1. Synthesis of intermediate compound (**5a-c**).

Synthesis of 5a: The title compound was obtained as an orange solid, (yield = 48%). ^{1}H NMR (500 MHz, $CDCl_3$): δ 7.76 (s, 1H), 7.67 (s, 1H), 7.39 (d, J = 6.5 Hz, 2H), 7.09 (d, J = 5.5 Hz, 4H), 6.90-6.85 (m, 6H), 4.14 (d, J = 7 Hz, 2H)), 3.81 (s, 6 H), 2.17-2.13 (m, 1H), 0.94-0.93 (m, 6H).

Synthesis of 5b: The title compound was obtained as an orange solid, (yield = 46%). ^{1}H NMR (500 MHz, $CDCl_3$): δ 7.74 (s, 1H), 7.65 (s, 1H), 7.37 (d, J = 9 Hz, 2H), 7.09 (d, J = 9 Hz, 4H), 6.89-6.85 (m, 6H), 4.26-4.16 (m, 2H)), 3.80 (s, 6H), 1.85-1.80 (m, 1H), 1.38-1.25 (m, 8H), 0.94-0.86 (m, 6H).

Synthesis of 5c: The title compound was obtained as an orange solid, (yield = 50%). ^{1}H NMR (500 MHz, $CDCl_3$): δ 7.75 (s, 1H), 7.66 (s, 1H), 7.38 (d, J = 9 Hz, 2H), 7.09 (d, J = 9 Hz, 4H), 6.89-6.85 (m, 6H), 4.21 (d, J = 7.5 Hz, 2H), 3.81 (s, 6H), 1.89-1.87 (m, 1H), 1.27-1.22 (m, 24H), 0.86-0.84 (m, 6H).

General procedure for the synthesis of 5-(8-(4-(bis(4-methoxyphenyl)amino)phenyl)-5-alkyl-4,6-dioxo-5,6-dihydro-4*H*-dithieno[3,2-*c*:2',3'-*e*]azepin-2-yl)thiophene-2-carbaldehyde (6a-c): Under anhydrous condition, $Pd(PPh_3)_4$ (0.05 equiv.) was added to a solution of 2-(4-(bis(4-methoxyphenyl)amino)phenyl)-8-bromo-5-alkyl-4*H*-dithieno[3,2-*c*:2',3'-*e*]azepine-4,6(5*H*)-dione (**5a-c**; 1 equiv.) and (5-(1,3-dioxolan-2-yl)thiophen-2-yl)tributylstannane (1 equiv.) in dry toluene. The resulting mixture was refluxed for 24 hours under nitrogen. After cooling to room temperature, the solvent was evaporated and the obtained residue was purified by column chromatography with ethyl acetate/hexanes as the eluent.

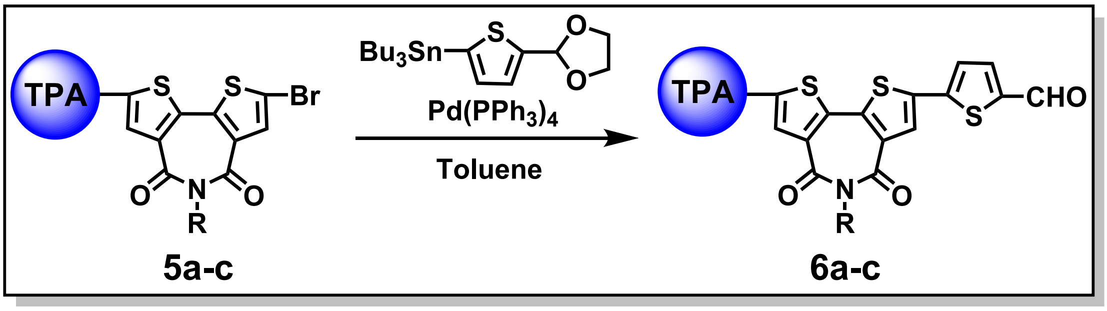

Scheme S2. Synthesis of intermediate compound (**6a-c**).

Synthesis of 6a: The title compound was obtained as a red solid, (yield = 80%). ^{1}H NMR (500 MHz, $CDCl_3$): δ 9.88 (s, 1H), 7.95 (s, 1H), 7.77 (s, 1H), 7.70 (d, J = 3 Hz, 1H), 7.42 (m, 2H), 7.31 (m, 1H), 7.06 (m, 4H)), 6.88 (m, 6H), 4.17 (d, J = 7 Hz, 2H)), 3.83 (s, 6H), 2.20-2.14 (m, 1H), 0.96-0.95 (m, 6H).

Synthesis of 6b: The title compound was obtained as a red solid, (yield = 77%). ^{1}H NMR (500 MHz, $CDCl_3$): δ 9.88 (s, 1H), 7.95 (s, 1H), 7.80 (s, 1H), 7.70 (d, J = 4 Hz, 1H), 7.40 (d, J = 8.5 Hz, 2H), 7.31 (d, J = 4 Hz, 1H), 7.09 (d, J = 8.5 Hz, 4H)), 6.90 (d, J = 8.5 Hz, 2H), 6.87 (d, J = 9 Hz, 4H), 4.29-4.19 (m, 2H)), 3.81 (s, 6H), 1.86-1.84 (m, 1H), 1.36-1.27 (m, 8H), 0.92-0.87 (m, 6H).

Synthesis of 6c: The title compound was obtained as a red solid, (yield = 79%). ^{1}H NMR (500 MHz, $CDCl_3$): δ 9.89 (s, 1H), 7.96 (s, 1H), 7.80 (s, 1H), 7.70 (d, J = 3.5 Hz, 1H), 7.40 (m, 2H), 7.31 (d, J = 3.5 Hz, 1H), 7.09 (m, 4H)), 6.88-6.86 (m, 6H), 4.25 (d, J = 7.5 Hz, 2H) , 3.81 (s, 6H), 1.91 (m, 1H), 1.23 (m, 24H), 0.85-0.83 (m, 6H).

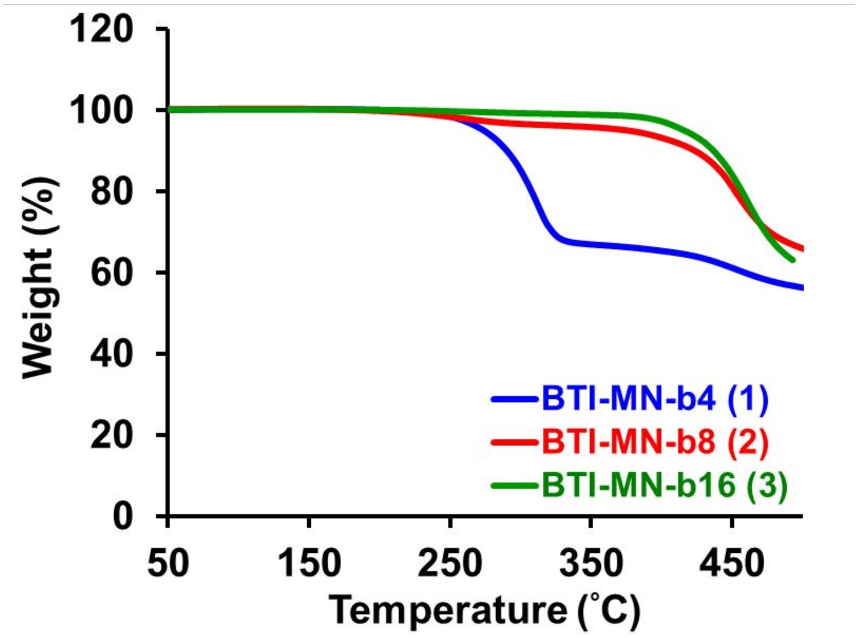

Figure S1. TGA curves of **BTI-MN** compounds.

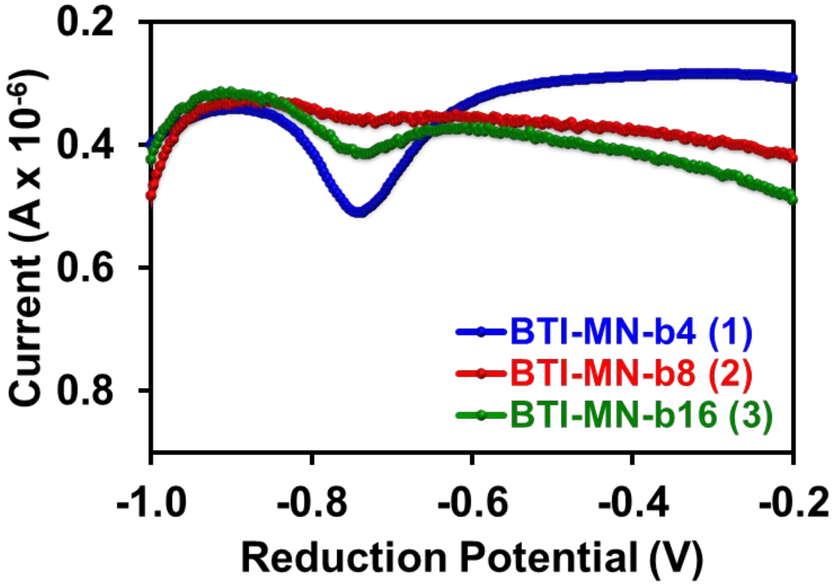

Figure S2. DPV curves of **BTI-MN** compounds in *o*-dichlorobenzene.

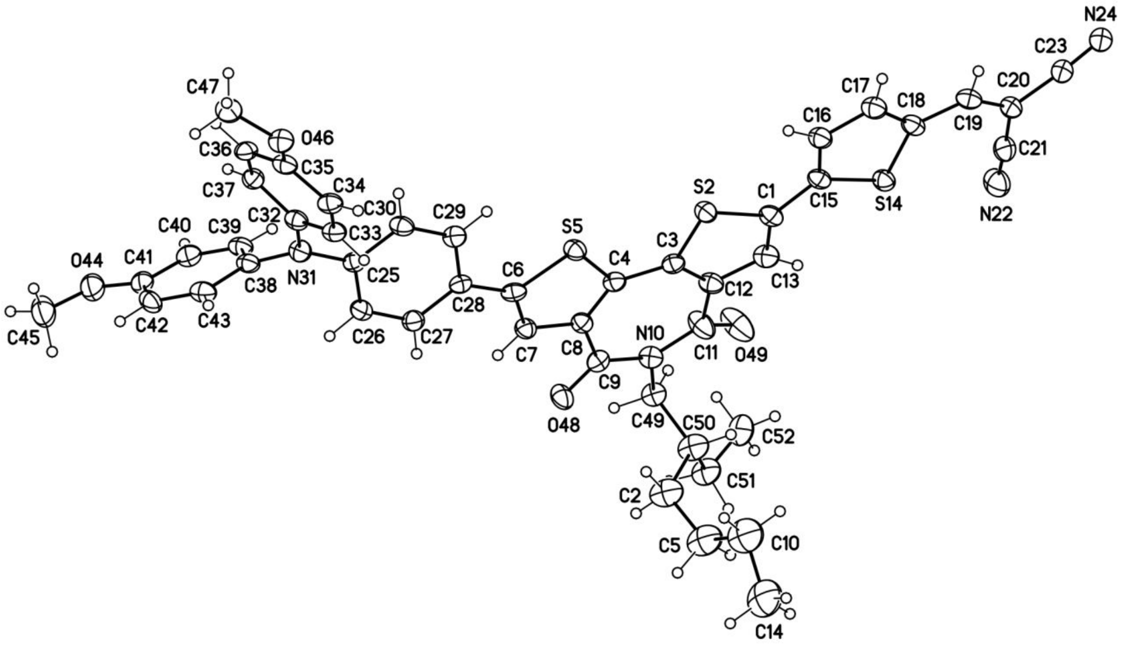

Figure S3. Perspective ORTEP drawing of the molecular structure of **BTI-MN-b8** (**2**).

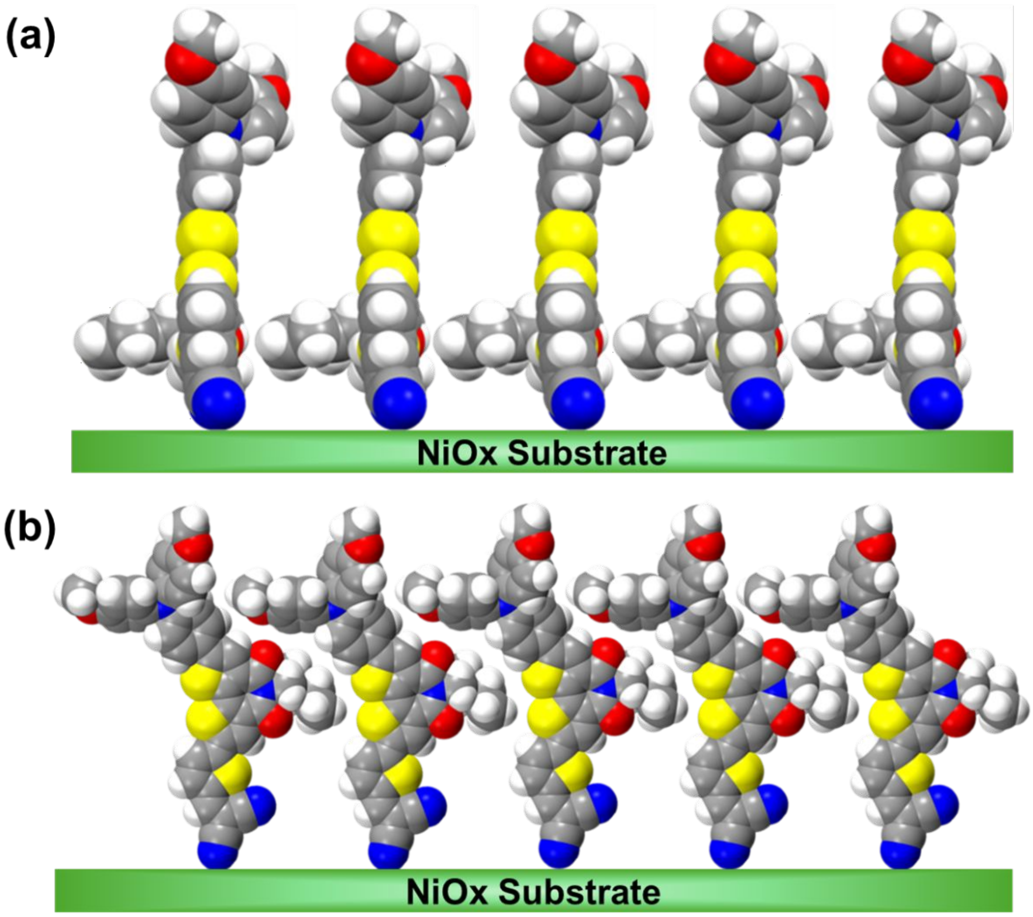

Figure S4. Expected packing pattern of **BTI-MN-b8** SAM molecule stands on NiOx/ITO substrate (a) with two legs (two CN) (side view); (b) with one leg (one CN) (front view).

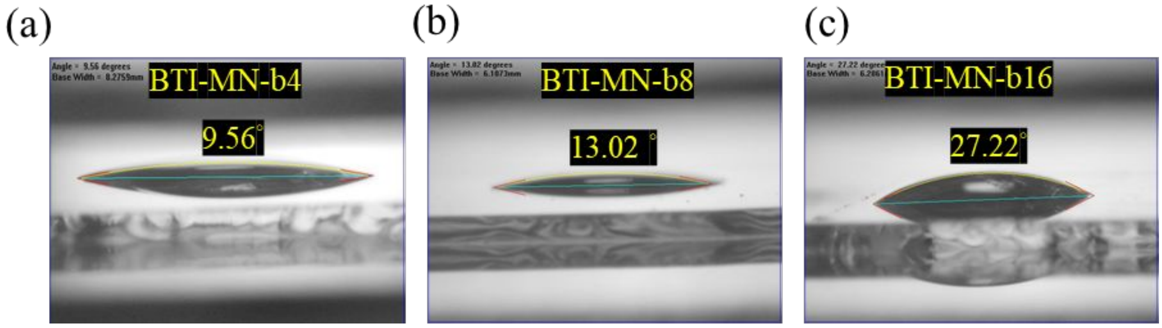

Figure S5. Precursor solution contact angles on (a) **BTI-MN-b4**, (b) **BTI-MN-b8** and (c) **BTI-MN-b16** films deposited on ITO substrate.

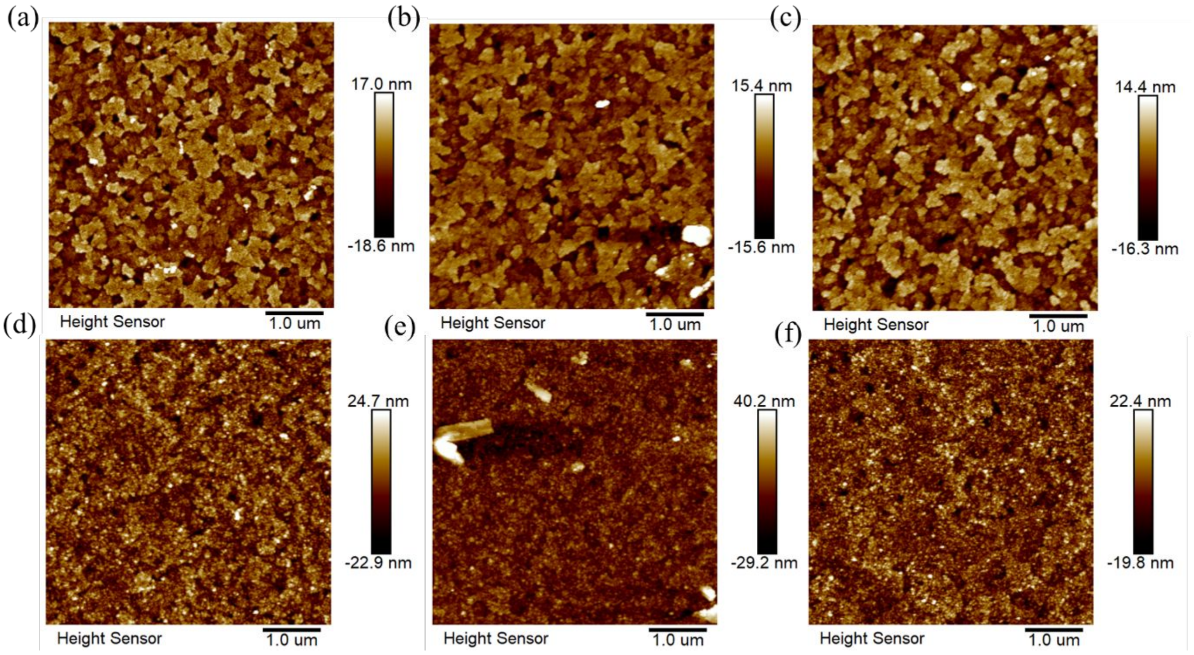

Figure S6. AFM images of (a) **BTI-MN-b4** on ITO, (b) **BTI-MN-b8** on ITO (c) **BTI-MN-b16** on ITO, (d) **BTI-MN-b4** on NiOx/ITO, (e) **BTI-MN-b8** on NiOx/ITO and (f) **BTI-MN-b16** on NiOx/ITO.

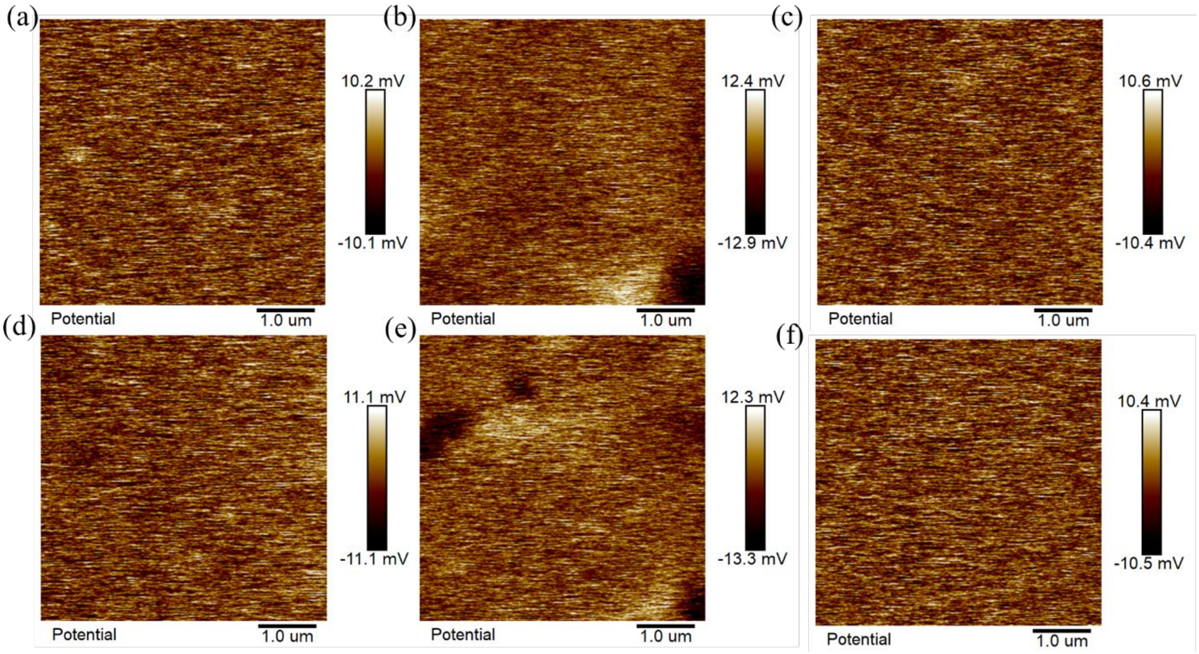

Figure S7. KPFM images of (a) **BTI-MN-b4** on ITO, (b) **BTI-MN-b8** on ITO (c) **BTI-MN-b16** on ITO, (d) **BTI-MN-b4** on NiOx/ITO, (e) **BTI-MN-b8** on NiOx/ITO and (f) **BTI-MN-b16** on NiOx/ITO.

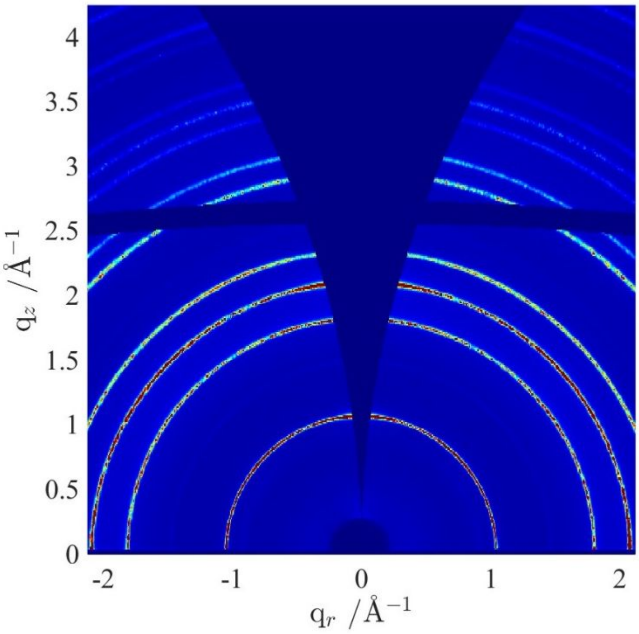

Figure S8. GIWAXS of tin perovskite made on the NiOx/BTI-MN-b8 film.

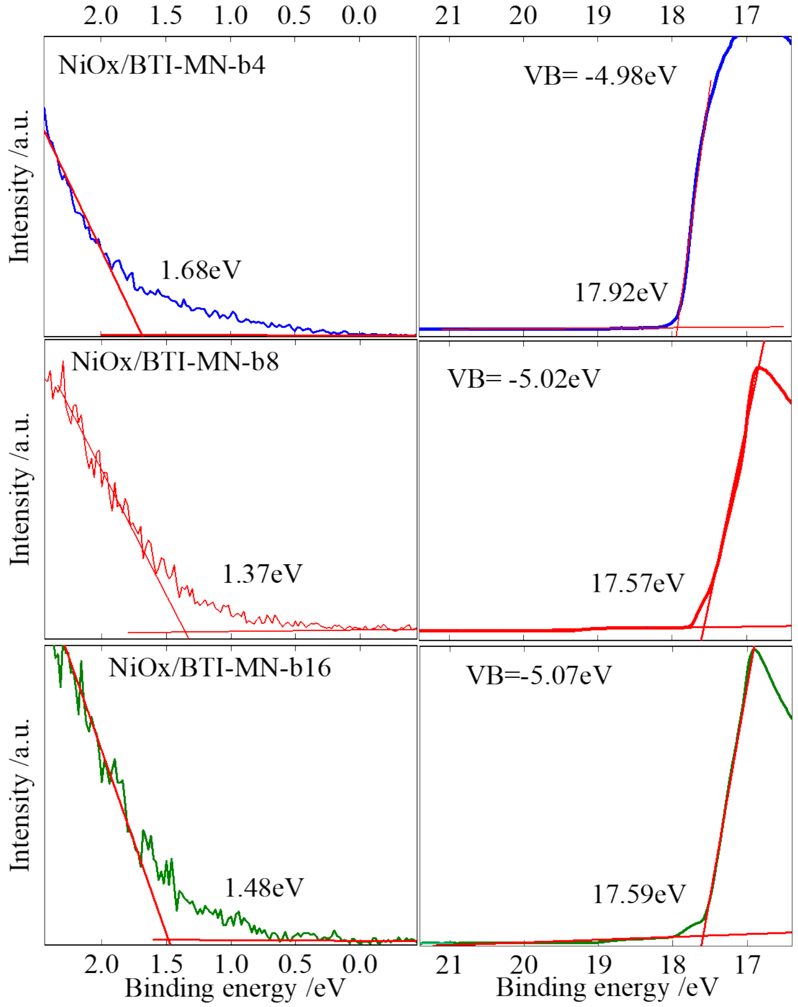

Figure S9. UPS raw data of the NiOx/SAMs films as indicated.

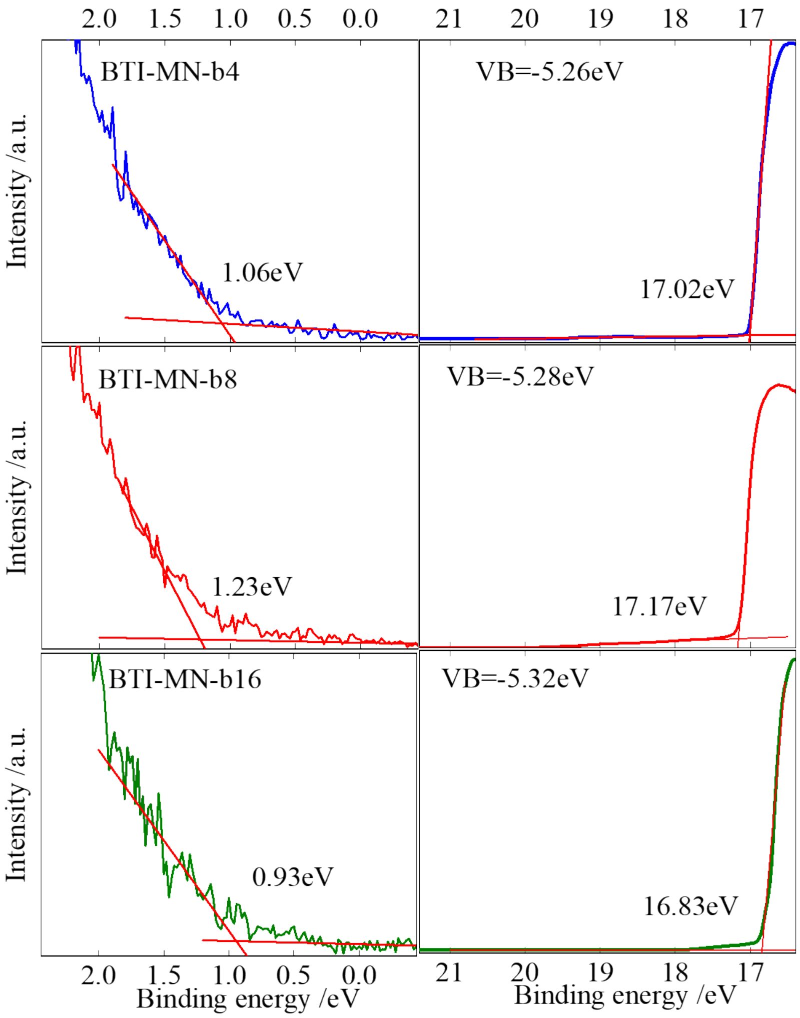

Figure S10. UPS raw data of varied SAM films (**BTI-MN-b4**, **BTI-MN-b8** and **BTI-MN-b16**) on ITO substrates.

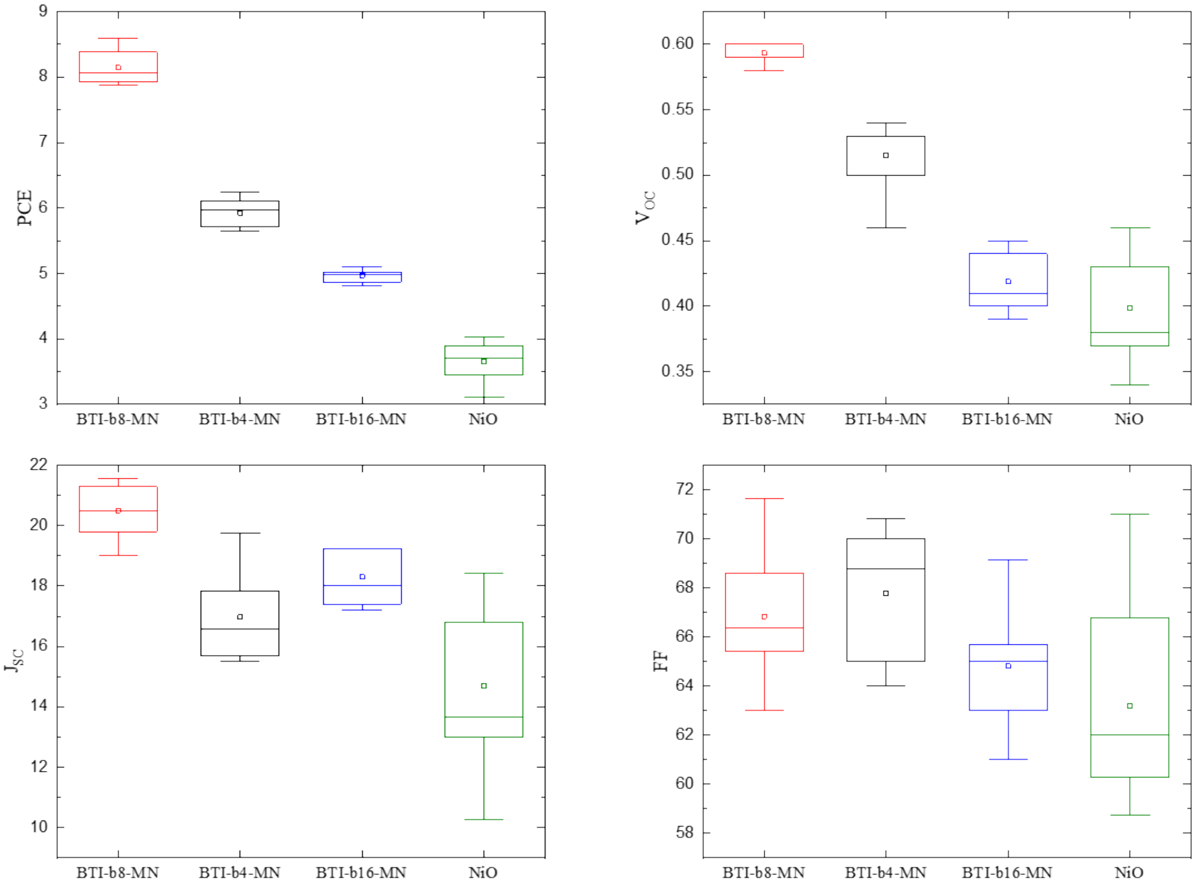

Figure S11. Boxplots of TPSCs for tin perovskite deposited on NiOx functionalized with varied SAMs as indicated.

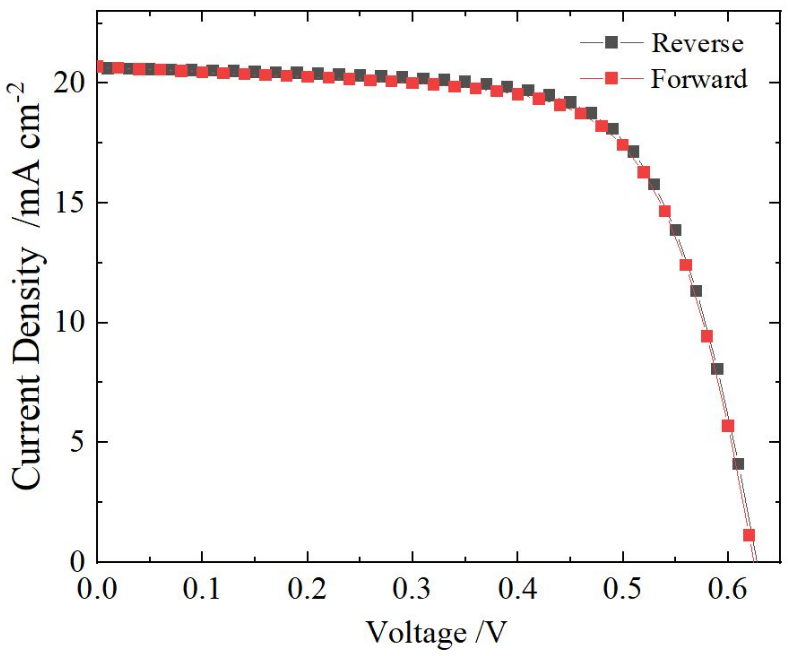

Figure S12. Effect of hysteresis of the NiOx/**BTI-MN-b8** device.

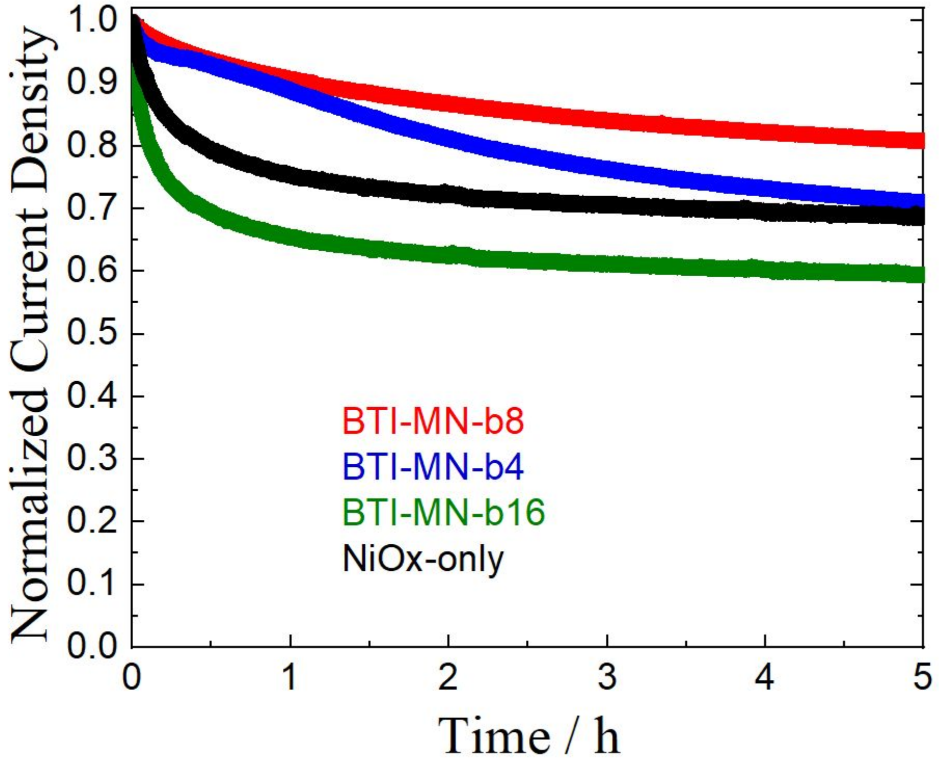

Figure S13. MPPT of TPSC with the NiOx only, NiOx/**BTI-MN-b4, NiOx/BTI-MN-b8** and **NiOx/BTI-MN-b16** device under one-sun irradiation.

Table S1. Summary of crystal structure data for **BTI-MN-b8** (**2**).

Identification code	240563lt2_auto
Empirical formula	$C_{46}H_{40}N_4O_4S_3$
Formula weight	809.00
Temperature/K	99.99(11)
Crystal system	orthorhombic
Space group	$Pca2_1$
a/Å	28.6609(14)
b/Å	16.0439(10)
c/Å	8.5986(5)
α/°	90
β/°	90
γ/°	90
Volume/Å^3	3953.9(4)
Z	4
ρ_{calc}g/cm^3	1.359
μ/mm^{-1}	2.122
F(000)	1696.0
Crystal size/mm^3	0.13 × 0.01 × 0.01
Radiation	Cu Kα (λ = 1.54184)
2Θ range for data collection/°	8.272 to 134.114
Index ranges	$-32 \leq h \leq 34$, $-18 \leq k \leq 19$, $-10 \leq l \leq 9$
Reflections collected	13825
Independent reflections	5631 [R_{int} = 0.0855, R_{sigma} = 0.0893]
Data/restraints/parameters	5631/220/594
Goodness-of-fit on F^2	1.015
Final R indexes [I>=2σ (I)]	R_1 = 0.0756, wR_2 = 0.1824
Final R indexes [all data]	R_1 = 0.1061, wR_2 = 0.2041
Largest diff. peak/hole / e Å^{-3}	0.61/-0.48
Flack parameter	0.14(5)

Crystallographic data (excluding structure factors) for the structure(s) reported in this paper have been deposited with the Cambridge Crystallographic Data Centre as supplementary publication no. **CCDC 2356550**.

Table S2. Sn^{2+} and Sn^{4+} proportions of tin perovskite based on NiOx, NiOx/BTI-MN-b4, NiOx/ BTI-MN-b8 and NiOx/ BTI-MN-b16 obtained from XPS measurements.

**Sample**	**NiOx**	**BTI-MN-b4**	**BTI-MN-b8**	**BTI-MN-b16**
Sn^{2+}	90.2%	95.9%	96.9%	91.1%
Sn^{4+}	9.8%	4.1%	3.1%	8.9%

Table S3. Fitting parameters of TCPSC for tin perovskites deposited on NiOx with varied SAMs.

Samples	τ_1/ns $(A_1\%)$	τ_2/ns $(A_2\%)$	Average Lifetime/ns
NiOx	3.17 (80.4%)	7.72 (19.6%)	4.87
NiOx/BTI-MN-b4	1.17 (98.5%)	4.49 (1.5%)	1.35
NiOx/BTI-MN-b8	1.08 (83.6%)	1.13 (16.3%)	1.08
NiOx/ BTI-MN-b16	2.01 (93.5%)	5.40 (6.5%)	2.54

Note: The average lifetime can be calculated with the equation: τ_{avg} = $(A_1\tau_1{}^2 + A_2\tau_2{}^2)/(A_1\tau_1+A_2\tau_2)$, where parameters A_1 and A_2 are the amplitude fractions for each decay component, and τ_1 and τ_2 represent the decay coefficients of the fits.

Table S4. Performance of TPSC based on NiOx with varied SAMs.

Devices	J_{SC}/mA cm^{-2}	V_{OC}/V	FF/%	PCE/%
NiOx	16.8	0.38	62.0	4.02
NiOx/BTI-MN-b4	18.2	0.52	68.3	6.53
NiOx/ BTI-MN-b8	19.8	0.61	71.4	8.62
NiOx/ BTI-MN-b16	17.9	0.43	66.7	5.14

Table S5. Photovoltaic parameters statistics of the **BTI-MN-b8** devices.

Device No.	Jsc/mA cm^{-2}	Voc/V	FF%	PCE%
1	21.4	0.6	66.39	8.59
2	21.55	0.6	65.3	8.46
3	20.9	0.6	66.6	8.38
4	20.75	0.6	66.55	8.29
5	21.29	0.6	64.4	8.27
6	20.34	0.6	66.2	8.07
7	21.35	0.6	66	8.5
8	20.5	0.59	66	7.97
9	21	0.59	63	7.97
10	19.8	0.58	68.6	7.92
11	20.1	0.6	65.4	7.87
12	19.8	0.58	68.6	7.87
13	21	0.57	64	7.81
14	21.5	0.57	65	7.99
15	21.3	0.57	64.5	7.9
16	21	0.59	63	7.88
17	21.23	0.59	62	7.74
18	21	0.57	64	7.81
19	19.64	0.56	68.4	7.58
Mean ± s.d.	20.81±0.61	0.59±0.01	65.47±1.88	8.05±0.28

Table S6. Photovoltaic parameters and statistics of the **BTI-MN-b4** devices.

Device No.	Jsc/mA cm^{-2}	Voc/V	FF%	PCE%
1	17.82	0.51	68.77	6.25
2	19.75	0.5	64	6.21
3	16.5	0.53	65.2	5.71
4	15.5	0.53	68.8	5.64
5	16.5	0.53	64	5.65
6	18.4	0.47	70.8	6.11
7	17.6	0.5	68	6.06
8	16	0.5	69.7	5.6
9	15.67	0.54	70.2	6
10	16.57	0.53	65	5.72
11	16.65	0.51	70	5.98
12	15.83	0.53	70.1	5.97
13	15.67	0.53	69.7	5.82
14	16.8	0.53	64.5	5.78
15	15.7	0.53	69.1	5.76
16	18.58	0.47	71	6.17
17	16	0.54	71	6.12
18	16.2	0.51	68.3	5.64
19	18	0.53	62	6
20	16.9	0.52	71	6.19
Mean ± s.d	16.83±1.2	0.52±0.02	67.9±2.83	5.9±0.22

Table S7. Photovoltaic parameters and statistics of the **BTI-MN-b16** devices.

Device No.	Jsc/mA cm^{-2}	Voc/V	FF%	PCE%
1	18.7	0.44	61.3	5.05
2	19.22	0.4	65.7	5.04
3	19.24	0.4	65.3	5.02
4	19.23	0.4	65.5	5.02
5	17.2	0.43	69.14	5.1
6	17.5	0.45	63	5.01
7	17.26	0.45	65.23	5.01
8	19.24	0.4	65	4.99
9	18	0.39	68.6	4.99
10	19.22	0.4	65	4.95
11	18	0.44	61.8	4.94
12	17.4	0.41	68	4.87
13	19.23	0.4	63	4.86
14	18	0.44	61	4.82
15	17.2	0.43	64.6	4.82
16	17	0.4	68	4.82
17	17.4	0.4	66	4.63
18	17.86	0.44	57.5	4.54
19	16	0.4	68	4.53
20	16.6	0.4	67	4.53
Mean ± s.d	18.05±0.95	0.42±0.02	64.82±3.1	4.9±0.17

Table S8. Photovoltaic parameters and statistics of the NiOx-only devices.

Device No.	Jsc/mA cm^{-2}	Voc/V	FF%	PCE%
1	15.8	0.34	60	3.21
2	12.9	0.43	71	3.98
3	17.1	0.37	61	3.89
4	16.45	0.38	59.8	3.76
5	16.2	0.37	62.3	3.75
6	13.02	0.41	70.8	3.73
7	13.6	0.46	61.2	3.71
8	13	0.456	61.5	3.65
9	18.1	0.34	59	3.63
10	13.55	0.42	62.3	3.59
11	12.13	0.41	69	3.45
12	15.25	0.35	60.3	3.28
13	13.65	0.38	62.12	3.26
14	10.27	0.45	66.8	3.11
15	9.96	0.44	68.77	3.03
16	15.37	0.35	54.23	2.99
17	14.4	0.36	57.43	2.96
18	11.88	0.34	67.21	2.74
19	10.65	0.44	55.5	2.6
20	15.8	0.34	60	3.21
Mean ± s.d	14.04±2.25	0.39±0.04	63.04±4.78	3.43±0.36

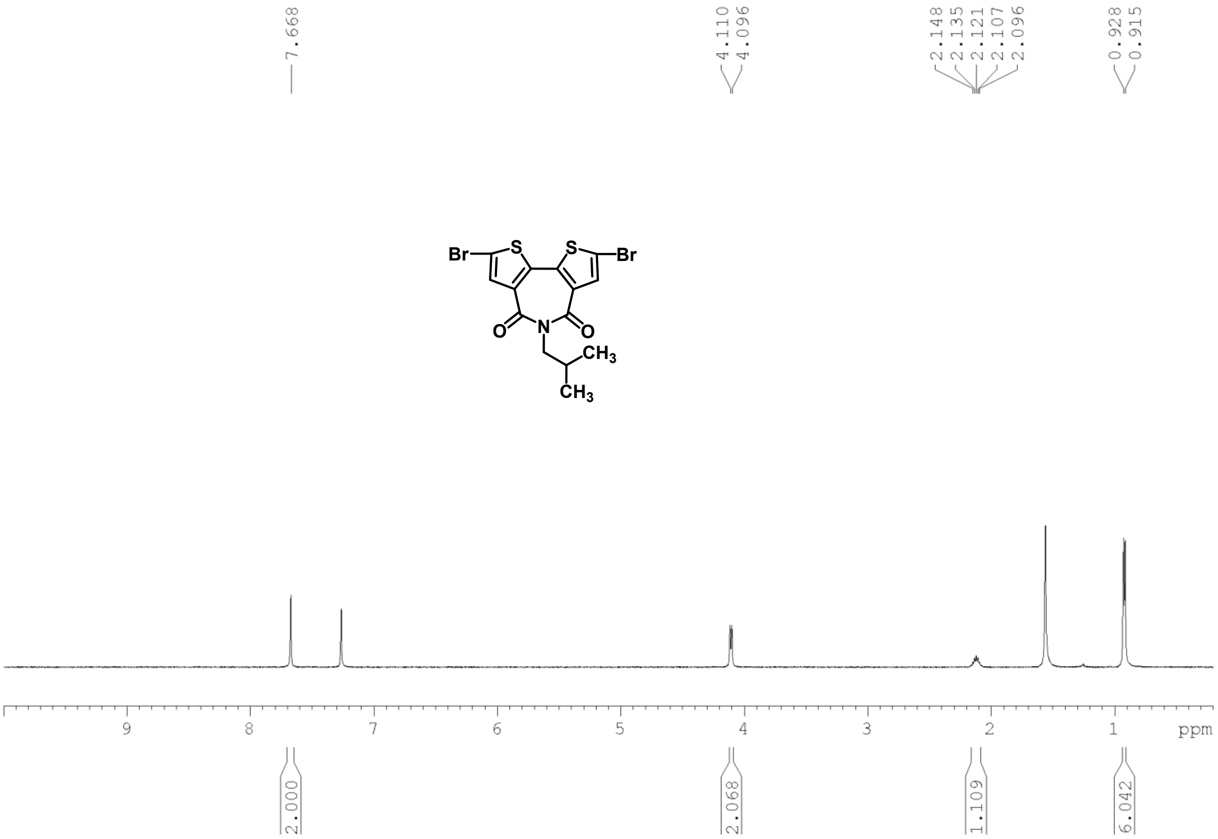

^{1}H NMR spectrum of **4a** in $CDCl_3$

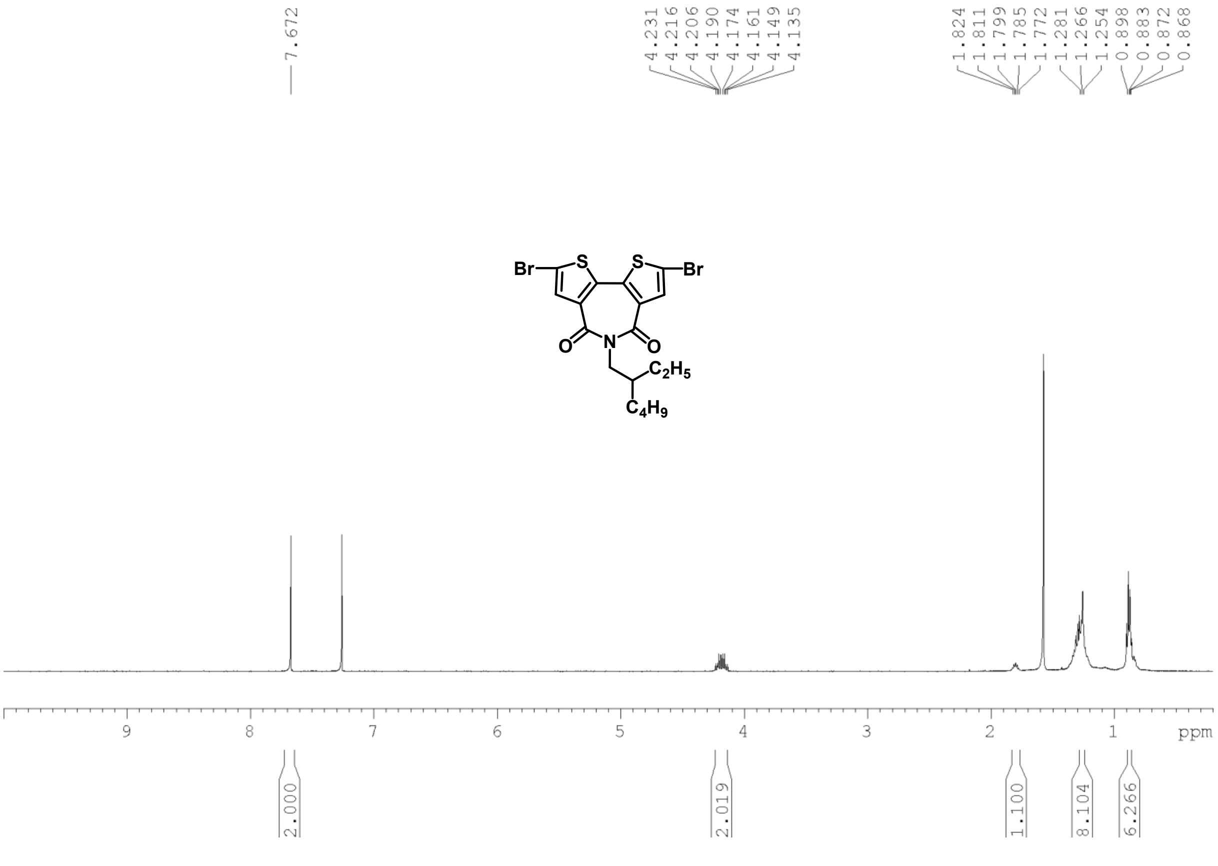

^{1}H NMR spectrum of **4b** in $CDCl_3$

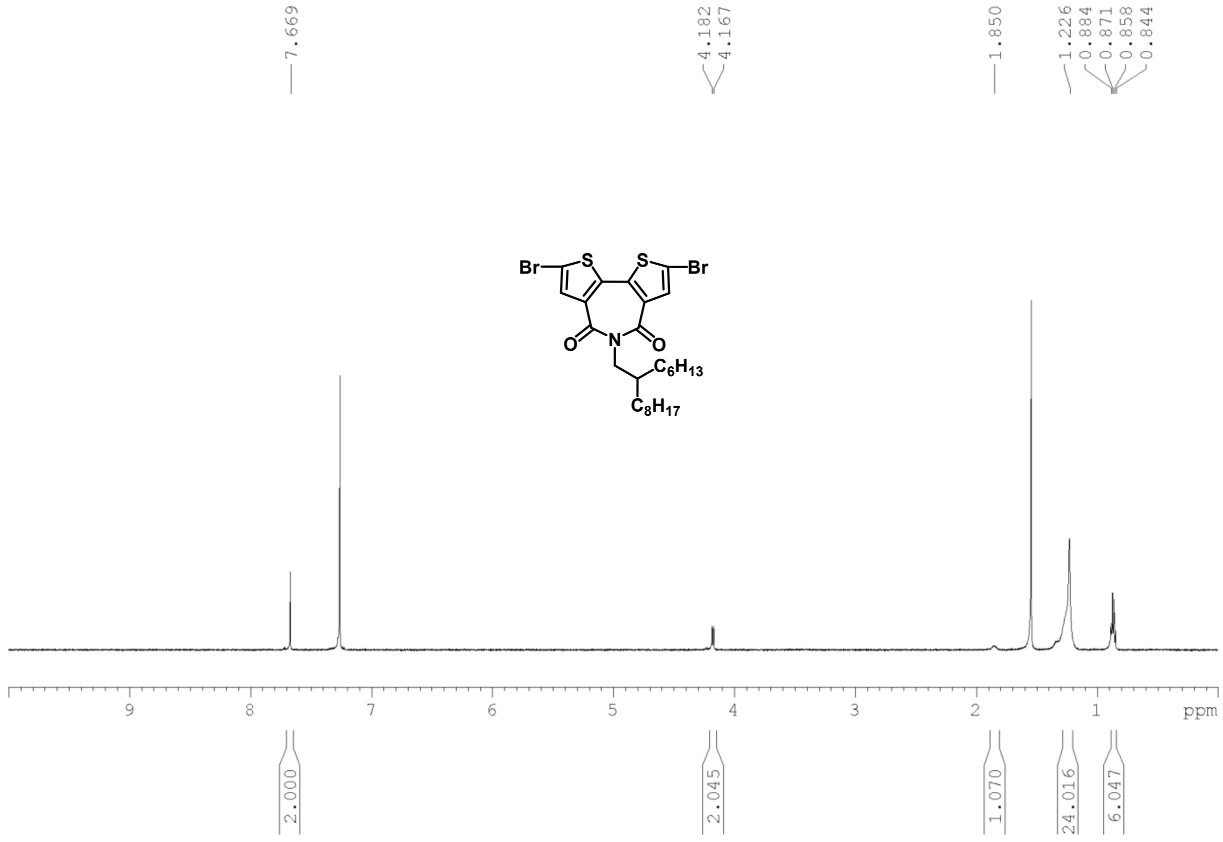

^{1}H NMR spectrum of **4c** in $CDCl_3$

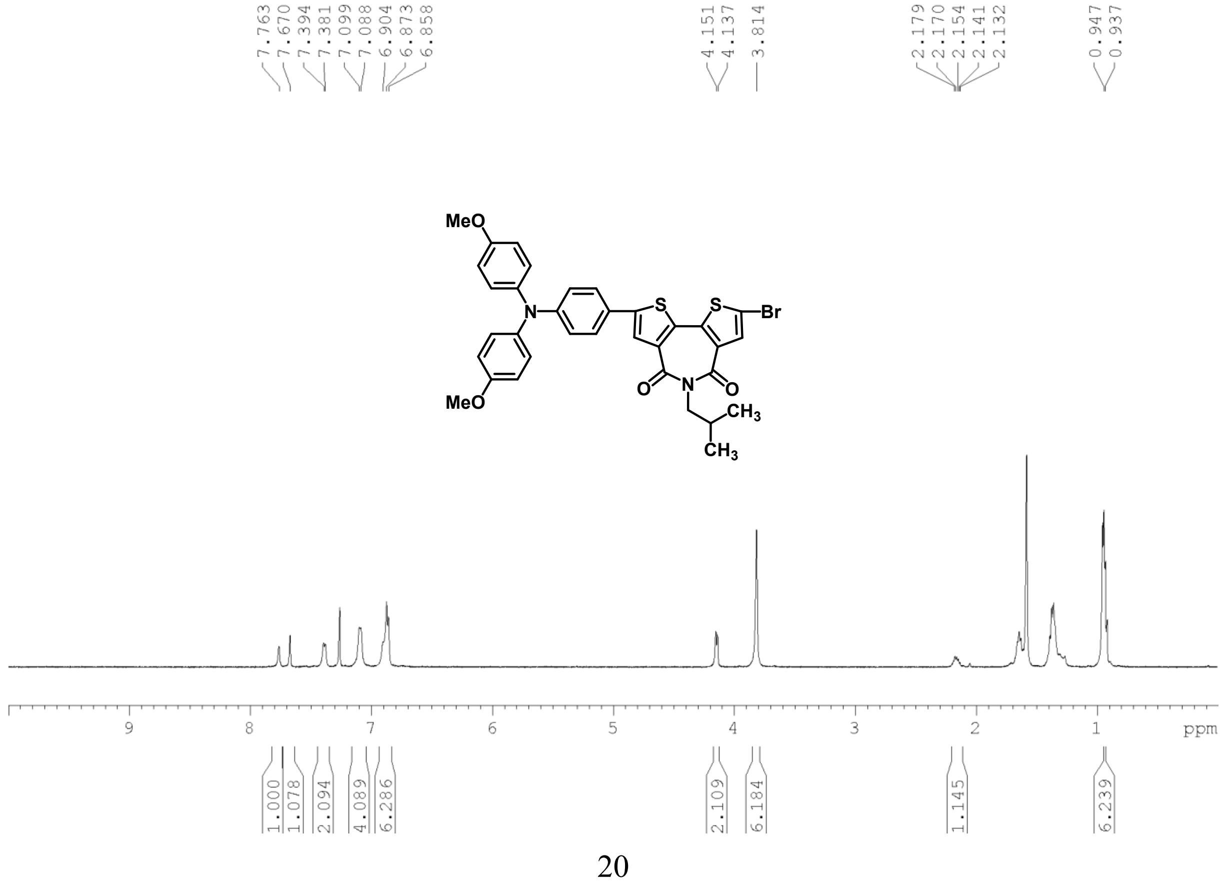

^{1}H NMR spectrum of **5a** in $CDCl_3$

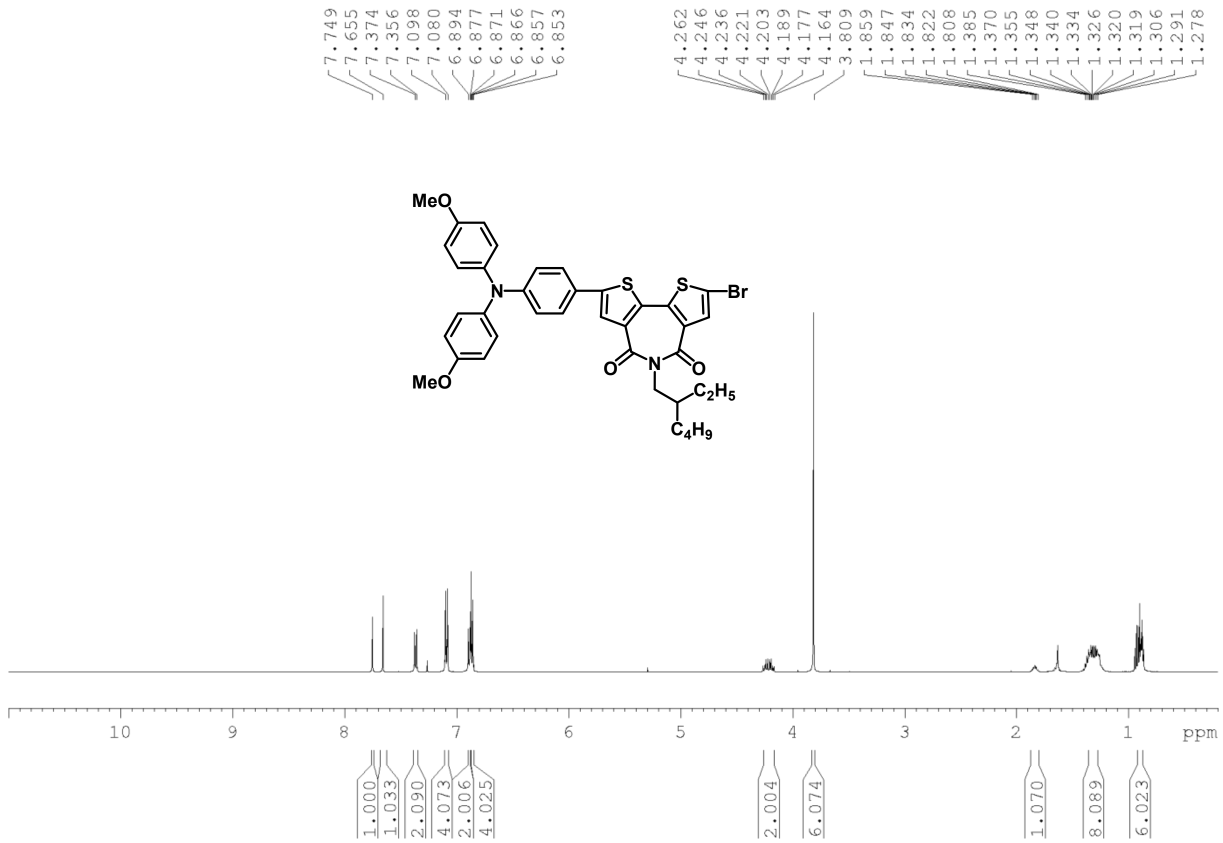

^{1}H NMR spectrum of **5b** in $CDCl_3$

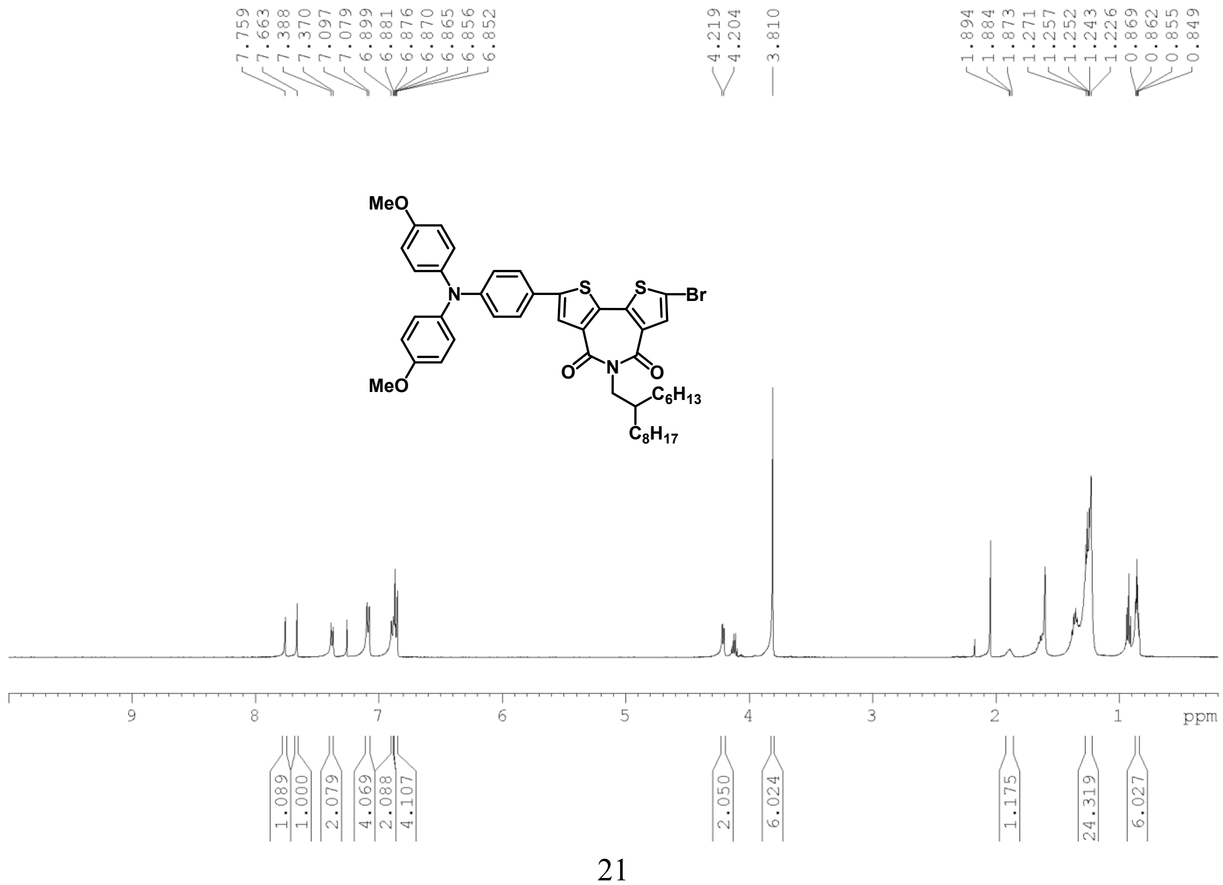

^{1}H NMR spectrum of **5c** in $CDCl_3$

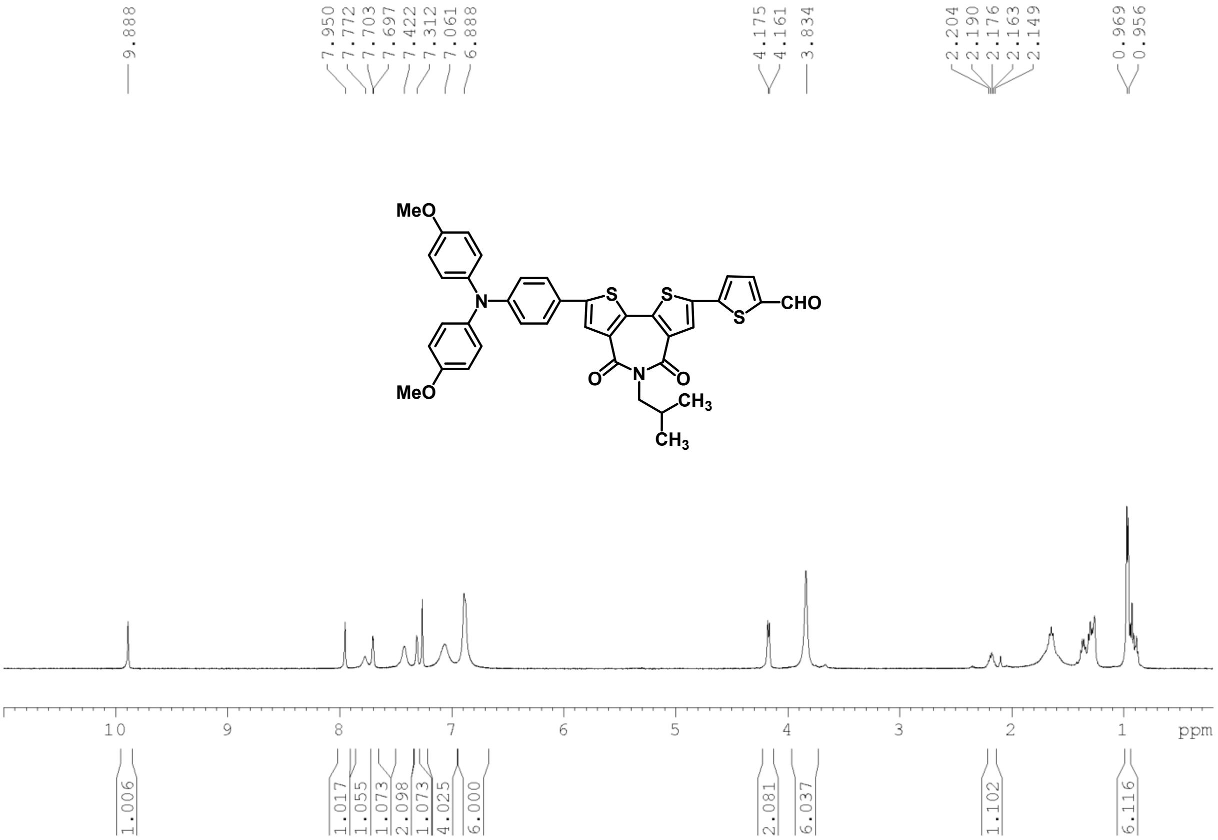

^{1}H NMR spectrum of **6a** in $CDCl_3$

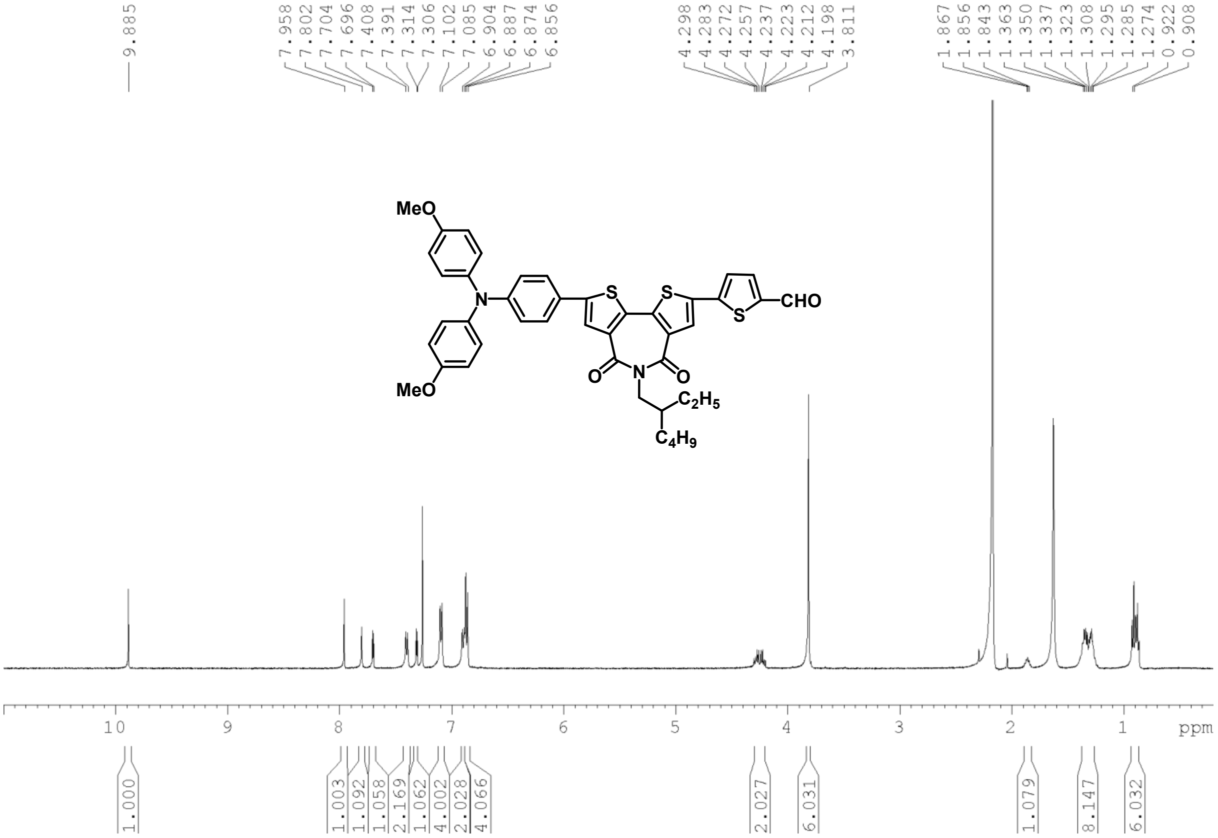

^1H NMR spectrum of **6b** in $CDCl_3$

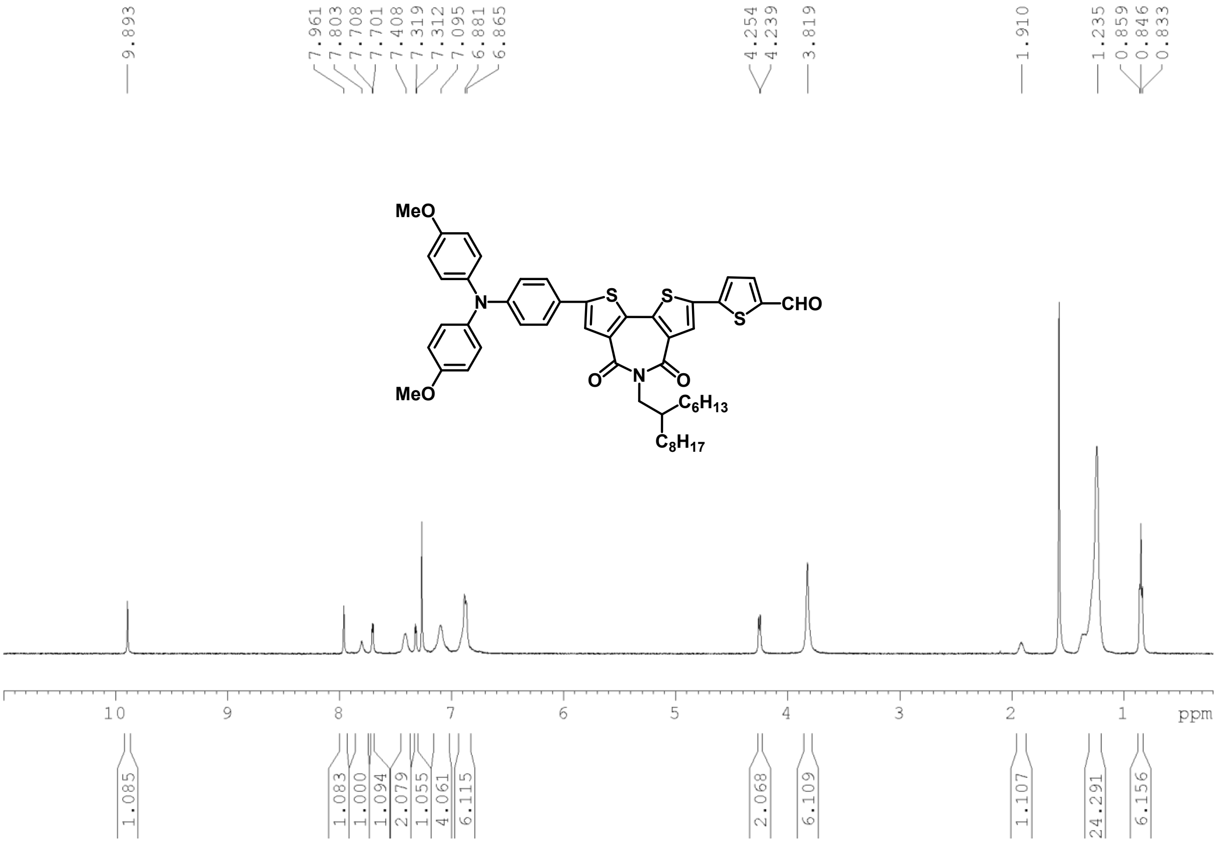

^1H NMR spectrum of **6c** in $CDCl_3$

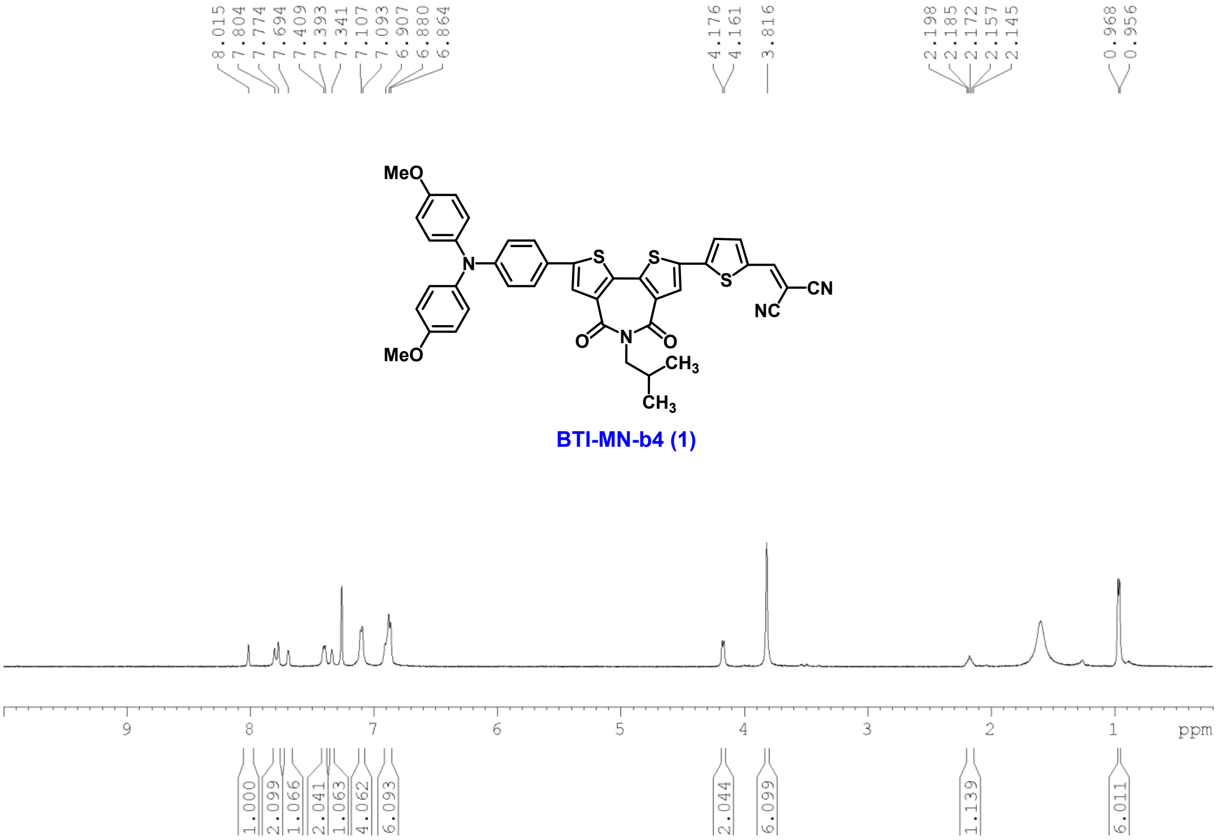

^{1}H NMR spectrum of **BTI-MN-b4** (**1**) in $CDCl_3$

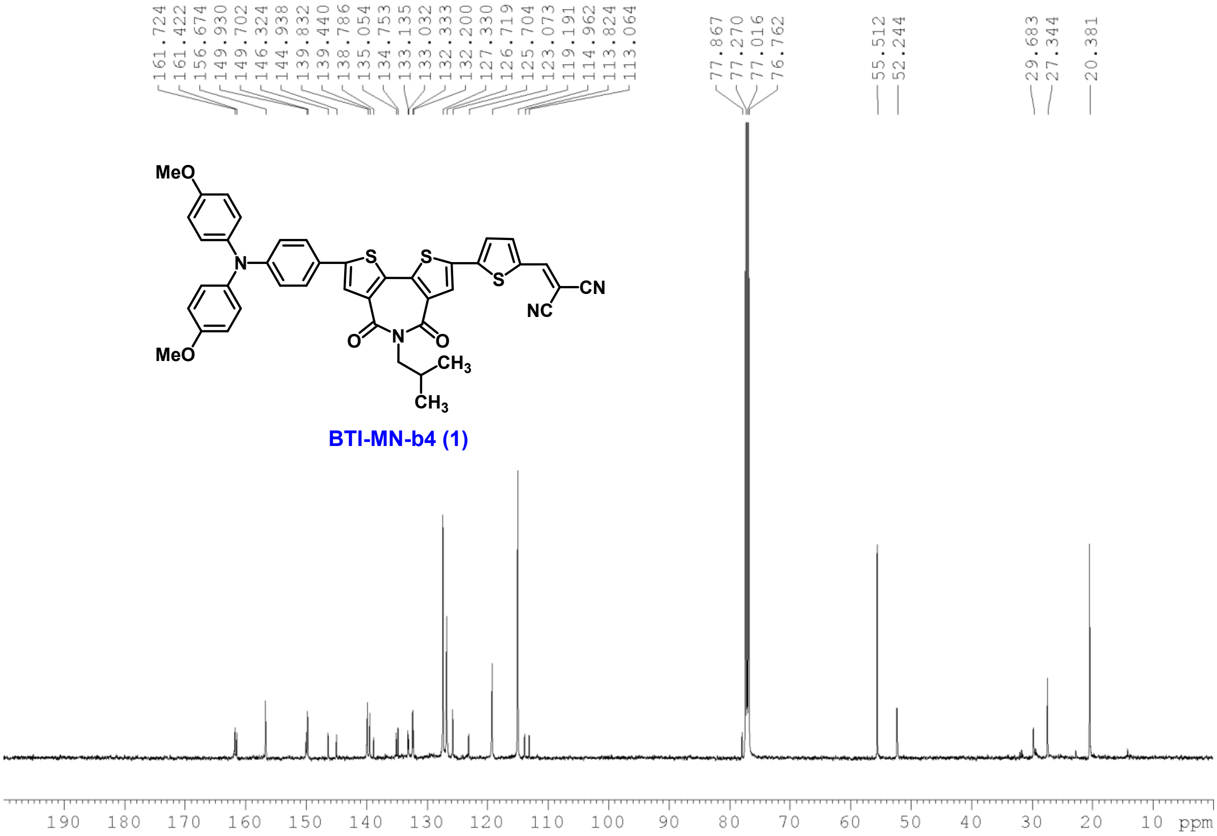

^{13}C NMR spectrum of **BTI-MN-b4** (**1**) in $CDCl_3$

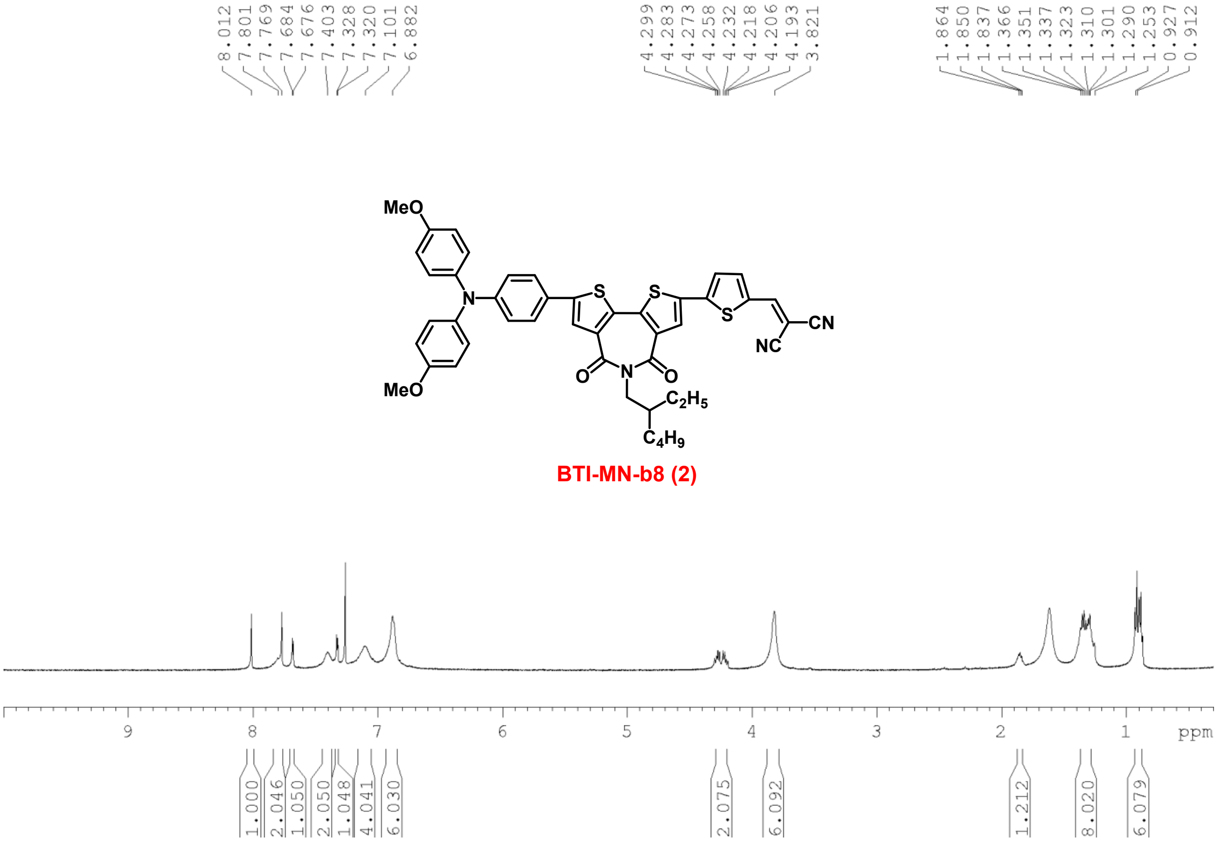

^{1}H NMR spectrum of **BTI-MN-b8** (**2**) in $CDCl_3$

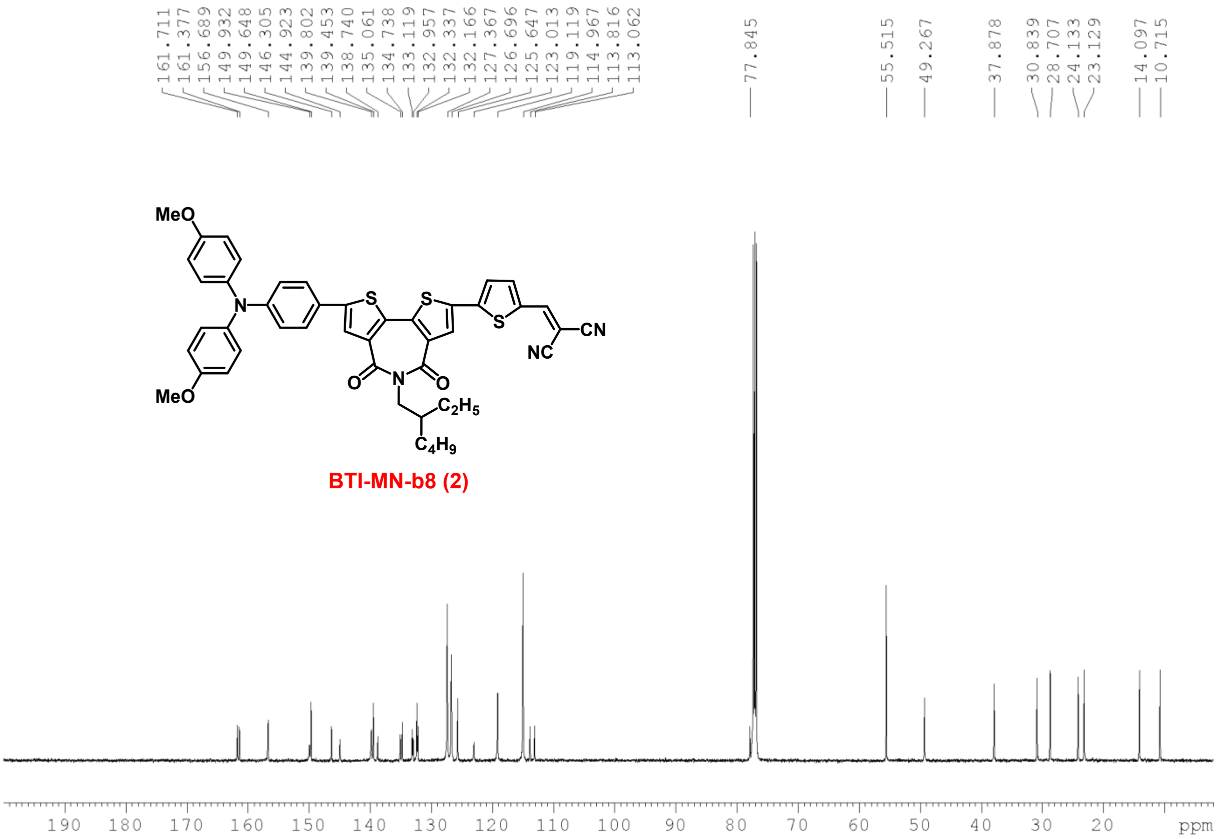

^{13}C NMR spectrum of **BTI-MN-b8** (**2**) in $CDCl_3$

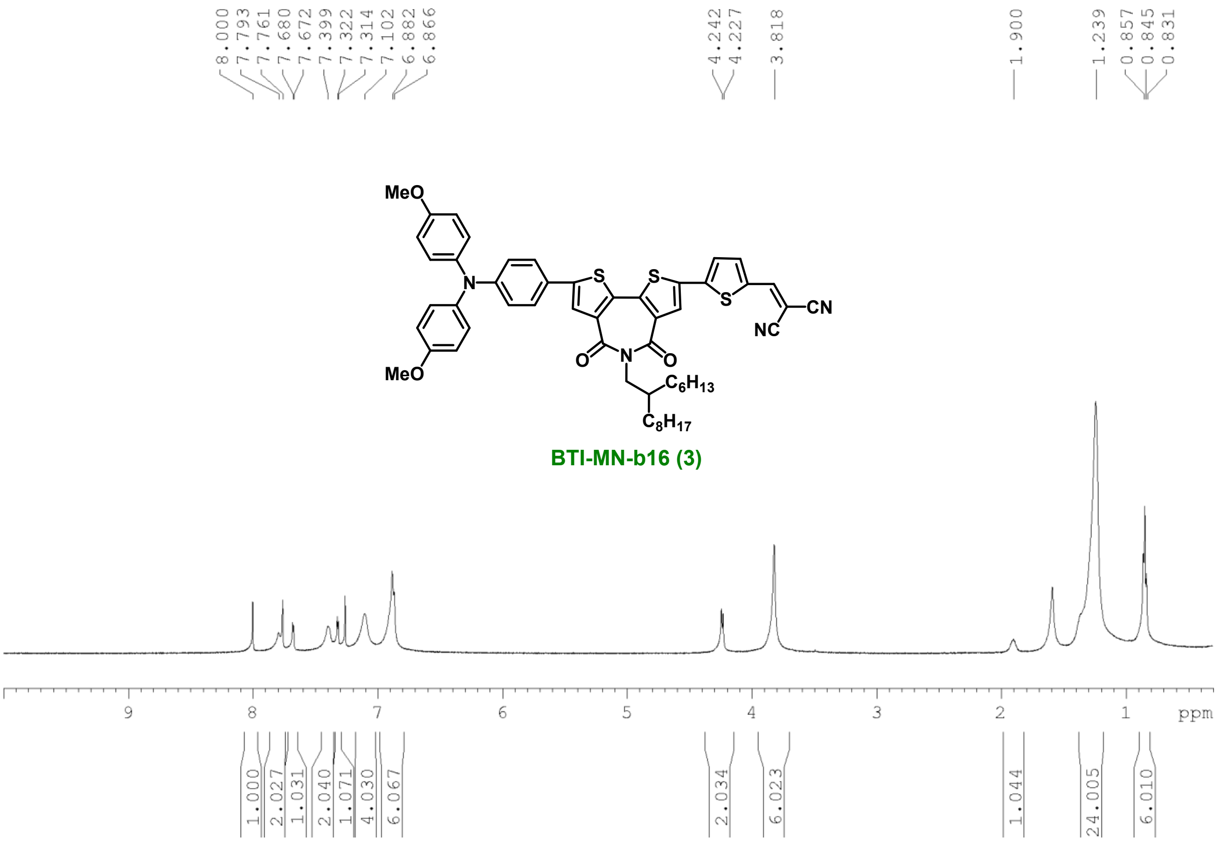

^{1}H NMR spectrum of **BTI-MN-b16** (**3**) in $CDCl_3$

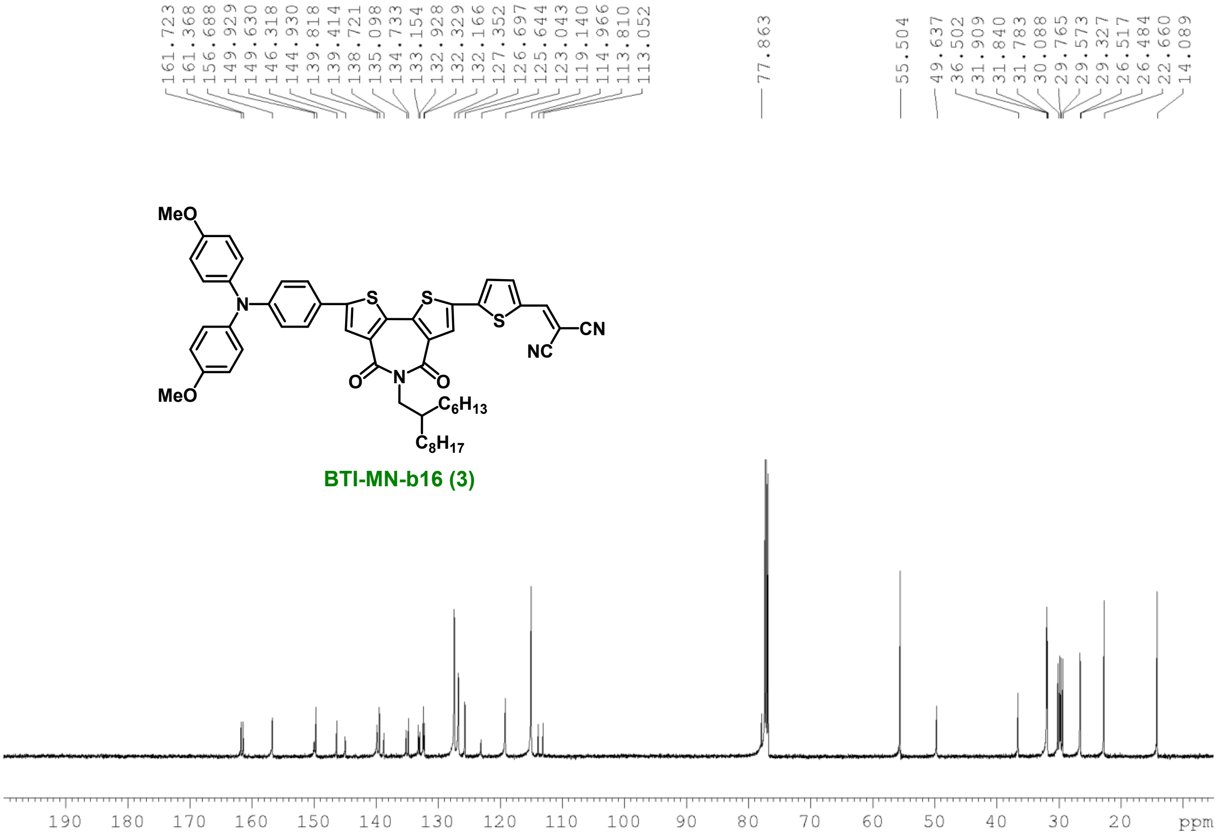

^{13}C NMR spectrum of **BTI-MN-b16** (**3**) in $CDCl_3$

Formula	Mass	Error	mSigma	DblEq	N rule	Electron Configuration
C 42 H 32 N 4 O 4 S 3	752.1580	3.4832	22.4081	29.00	ok	odd

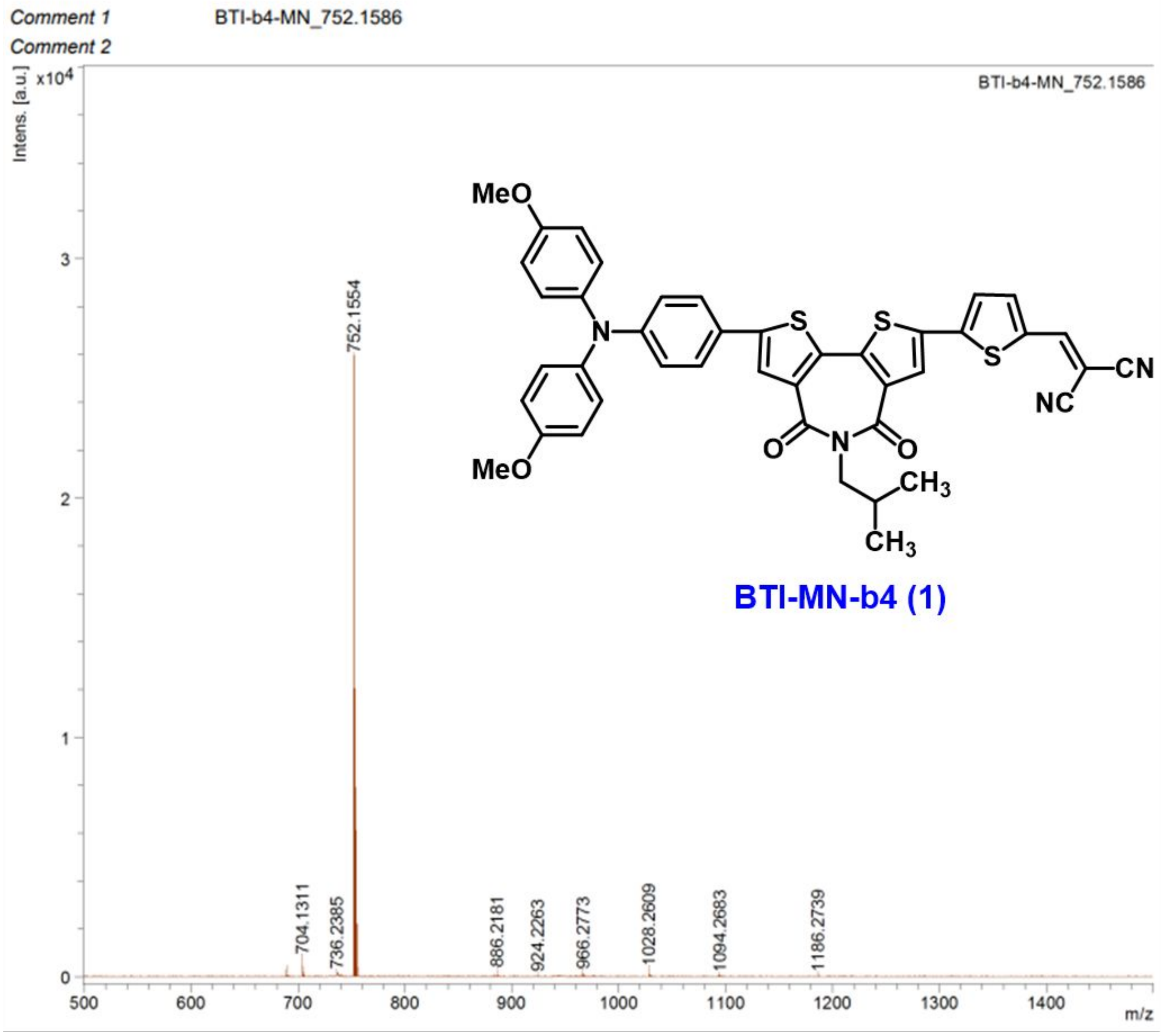

HRMS spectrum of **BTI-MN-b4** (**1**)

Formula	Mass	Error	mSigma	DblEq	N rule	Electron Configuration
C 46 H 40 N 4 O 4 S 3	808.2206	4.5529	50.1514	29.00	ok	odd

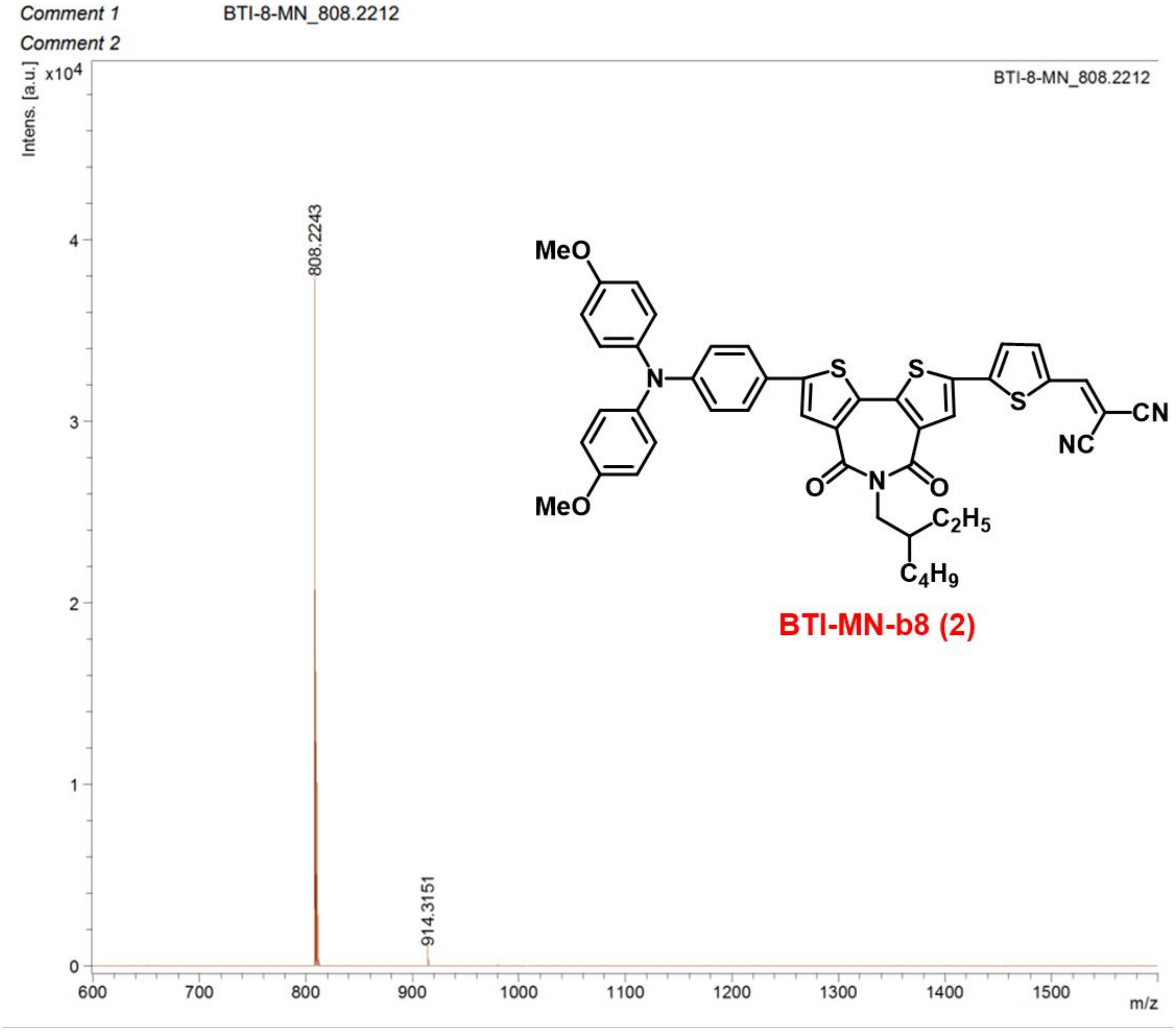

HRMS spectrum of **BTI-MN-b8** (**2**)

Formula	Mass	Error	mSigma	DblEq	N rule	Electron Configuration
C 54 H 56 N 4 O 4 S 3	920.3458	3.7804	115.0838	29.00	ok	odd

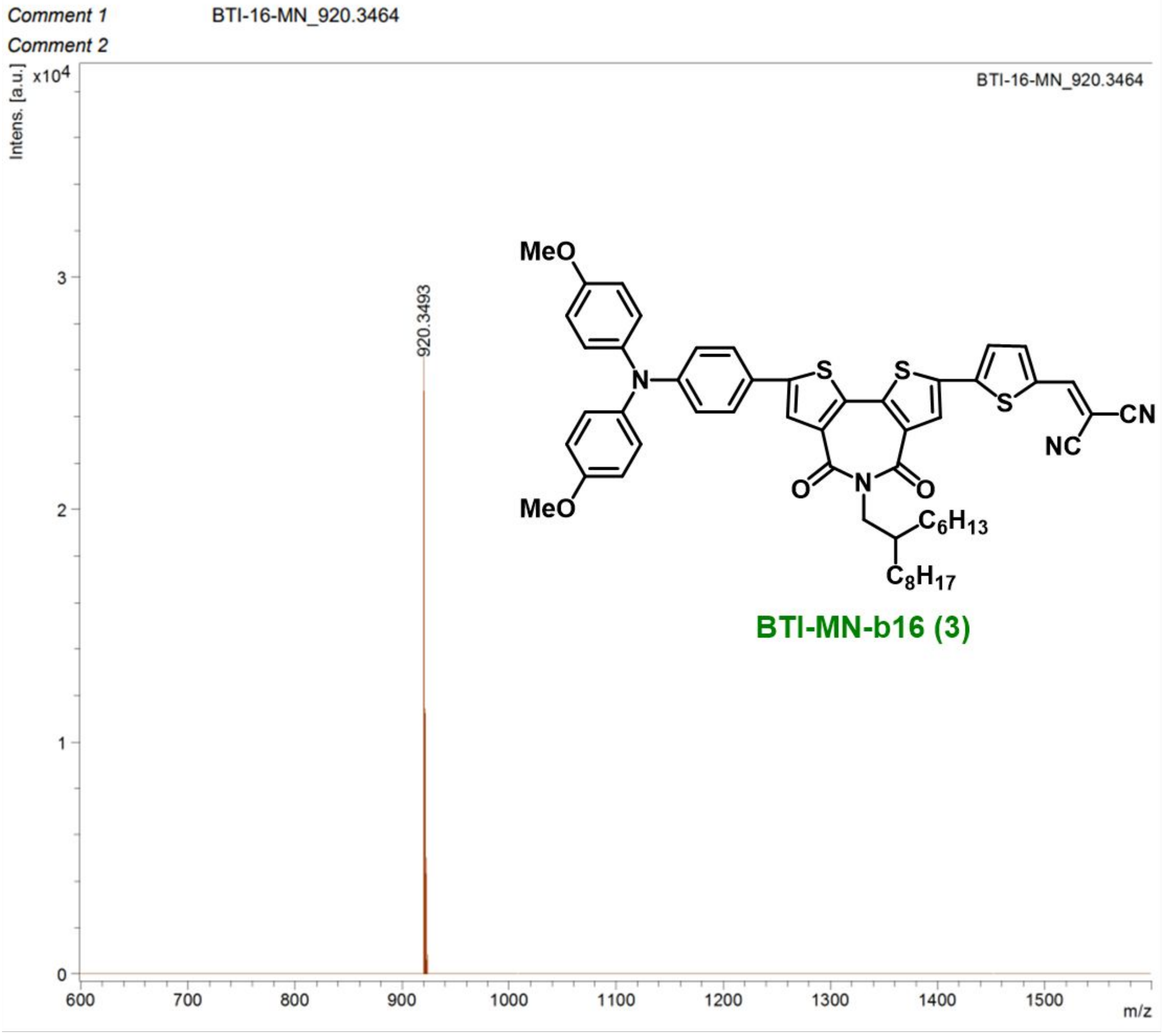

HRMS spectrum of **BTI-MN-b16** (**3**)
